# Heterogeneity of Melanoma Cell Responses to Sleep Apnea-Derived Plasma Exosomes and to Intermittent Hypoxia

**DOI:** 10.3390/cancers13194781

**Published:** 2021-09-24

**Authors:** Abdelnaby Khalyfa, Wojciech Trzepizur, Alex Gileles-Hillel, Zhuanhong Qiao, David Sanz-Rubio, José M. Marin, Miguel A. Martinez-Garcia, Francisco Campos-Rodriguez, Isaac Almendros, Ramon Farre, Manuel Sanchez-de-la-Torre, Francisco García-Río, David Gozal

**Affiliations:** 1 Department of Child Health and the Child Health Research Institute, University of Missouri School of Medicine, Columbia, MO 65201, USA; khalyfaa@health.missouri.edu (A.K.); qiaoz@health.missouri.edu (Z.Q.); 2 Department of Respiratory and Sleep Medicine, INSERM Unit 1063, Angers University Hospital, 49000 Angers, France; wotrzepizur@chu-angers.fr; 3 Pediatric Pulmonology and Sleep Unit, Hadassah Medical Center, Jerusalem 9987500, Israel; alex.gileles@mail.huji.ac.il; 4 Faculty of Medicine, The Hebrew University of Jerusalem, Jerusalem 9987500, Israel; 5 Translational Research Unit, Hospital Universitario Miguel Servet, IISAragon, Department of Medicine, University of Zaragoza, 50002 Zaragoza, Spain; dsanz@iisaragon.es (D.S.-R.); jmmarint@unizar.es (J.M.M.); 6 CIBER de Enfermedades Respiratorias, ISCIII, 28012 Madrid, Spain; martinez_miggar@gva.es (M.A.M.-G.); franciscoj.campos.sspa@juntadeandalucia.es (F.C.-R.); isaac.almendros@ub.edu (I.A.); rfarre@ub.edu (R.F.); manuel.sanchez@udl.cat (M.S.-d.-l.-T.); francisco.garcia@uam.es (F.G.-R.); 7 Pneumology Department, University and Polytechnic la Fe Hospital, 46026 Valencia, Spain; 8 Respiratory Department, Hospital Universitario de Valme, 41014 Sevilla, Spain; 9 Unitat de Biofísica i Bioenginyeria, Facultat de Medicina i Ciencies de la Salut, Universitat de Barcelona-Institut Investigacions Biomediques August Pi Sunyer, 08036 Barcelona, Spain; 10 Group of Precision Medicine in Chronic Diseases, Hospital Arnau de Vilanova-Santa Maria, IRBLleida, Department of Nursing and Physiotherapy, Faculty of Nursing and Physiotherapy, University of Lleida, 25198 Lleida, Spain; 11 Respiratory Department, Hospital Universitario La Paz-IdiPAZ, Universidad Autónoma de Madrid, 28046 Madrid, Spain

**Keywords:** sleep apnea, positive airway pressure, CPAP, exosomes, melanoma, miRNAs, AMPK, CRL-1424, CRL-1619, CRL-1675, intermittent hypoxia

## Abstract

**Simple Summary:**

Obstructive sleep apnea has been implicated in deleterious effects on cancer incidence and outcomes. Among the cancers identified as affected by sleep apnea, melanoma has consistently emerged as particularly susceptible. Furthermore, evidence suggests that exosomes, microparticles that originate from all cells and contain a variety of biologically active molecules such as microRNAs, can modify cancer cell properties. Here, we examined whether exosomes from sleep apnea patients can alter melanoma cell properties, and whether such effects were attributable to selected microRNA candidates, and affected melanoma cells harboring different mutations differently. In these experiments, the divergent responses of three melanoma cell lines to exosomes from sleep apnea patients before and after treatment indicated that STK11 gene mutations in melanoma cells make them particularly responsive to sleep apnea by altering cellular metabolism, and that specific microRNAs appear to underlie such effects.

**Abstract:**

Obstructive sleep apnea (OSA) is associated with increased cutaneous melanoma incidence and adverse outcomes. Exosomes are secreted by most cells, and play a role in OSA-associated tumor progression and metastasis. We aimed to study the effects of plasma exosomes from OSA patients before and after adherent treatment with continuous positive airway pressure (CPAP) on melanoma cells lines, and also to identify exosomal miRNAs from melanoma cells exposed to intermittent hypoxia (IH) or normoxia. Plasma-derived exosomes were isolated from moderate-to-severe OSA patients before (V1) and after (V2) adherent CPAP treatment for one year. Exosomes were co-incubated with three3 different melanoma cell lines (CRL 1424; CRL 1619; CRL 1675) that are characterized by genotypes involving different mutations in BRAF, STK11, CDKN2A, and PTEN genes to assess the effect of exosomes on cell proliferation and migration, as well as on pAMK activity in the presence or absence of a chemical activator. Subsequently, CRL-1424 and CRL-1675 cells were exposed to intermittent hypoxia (IH) and normoxia, and exosomal miRNAs were identified followed by GO and KEG pathways and gene networks. The exosomes from these IH-exposed melanoma cells were also administered to THP1 macrophages to examine changes in M1 and M2 polarity markers. Plasma exosomes from V1 increased CRL-1424 melanoma cell proliferation and migration compared to V2, but not the other two cell lines. Exposure to CRL-1424 exosomes reduced pAMPK/tAMPK in V1 compared to V2, and treatment with AMPK activator reversed the effects. Unique exosomal miRNAs profiles were identified for CRL-1424 and CRL-1675 in IH compared to normoxia, with six miRNAs being regulated and several KEGG pathways were identified. Two M1 markers (CXCL10 and IL6) were significantly increased in monocytes when treated with exosomes from IH-exposed CRL-1424 and CRL-1625 cells. Our findings suggest that exosomes from untreated OSA patients increase CRL-1424 melanoma malignant properties, an effect that is not observed in two other melanoma cell lines. Exosomal cargo from CRL-1424 cells showed a unique miRNA signature compared to CRL-1675 cells after IH exposures, suggesting that melanoma cells are differentially susceptible to IH, even if they retain similar effects on immune cell polarity. It is postulated that mutations in STK-11 gene encoding for the serine/threonine kinase family that acts as a tumor suppressor may underlie susceptibility to IH-induced metabolic dysfunction, as illustrated by CRL-1424 cells.

## 1. Introduction

Obstructive sleep apnea (OSA) is a frequent disease that occurs among individuals of all ages, and has recently been associated with an increased risk of cancer incidence and mortality [1,2]. Among the potential tumors exhibiting significant associations with OSA related to incidence and outcomes [3,4,5,6,7,8], cutaneous melanoma has emerged as one of the malignancies that most consistently emerges in epidemiological studies [9], while such association may not be as reliably present for other tumor types [8,10,11]. Previously, in murine models we have collected evidence supporting the conceptual framework that sleep apnea, i.e., intermittent hypoxia (IH) and sleep fragmentation (SF) could causally modify cancer-related biological processes. These observations prompted us to hypothesize that selected types of solid cancers may be particularly responsive to the underlying presence of OSA, and that their incidence or outcomes may be adversely affected in OSA patients [12,13]. 

In the United States, cutaneous melanoma rates (the most aggressive skin tumor type) have continuously increased over the last two decades, and continue to increase. In the framework of OSA, in addition to the aforementioned epidemiological evidence, several studies using animal models to study melanoma tumor growth [14,15], metastasis [12] and potential interactions with obesity [16] confirmed that intermittent hypoxia mimicking the oscillations in oxygen tension that characterize OSA induce significant changes in the biological properties of melanoma cells, thereby promoting accelerated proliferation, invasion and resistance to therapy [12,16,17,18,19,20,21]. However, it has also become apparent that there is considerable variability in the response to hypoxia by any type of malignant tumor, and that such heterogeneity may account for the conflicting findings in epidemiological studies attempting to link between OSA and cancer [22]. Thus, improved understanding of the molecular and cellular processes underlying melanoma plasticity and heterogeneity is of paramount importance.

Tumor cells and adjoining cells within the tumor microenvironment can communicate by a variety of processes involving cell–cell interactions as well as paracrine mechanisms [23,24]. More recently, exosomes have been suggested as one of the key mechanisms of intercellular communication in the tumor microenvironment, in light of their ubiquitous presence in bodily fluids, as well as their ability to reliably deliver genetic messages between cells, ultimately altering the phenotype of the target cells [25,26,27,28,29,30,31,32,33,34]. In previous studies, we showed that both exosomes derived from plasma of mice exposed to IH or SF emulating the perturbations of OSA, and exosomes from plasma samples of patients with OSA altered tumor growth and enhanced specific properties of cancer tumor cell cultures [27,28]. In this study, we hypothesized that circulating exosomes from patients with OSA will induce changes in naïve human melanoma cells in vitro, but that such effects may vary among melanoma cell lines, and that such differences will also be reflected in the exosomal miRNA cargo originating from melanoma cells exposed to IH in vitro. Furthermore, we aimed to assess the effect of exosomes derived from IH-exposed melanoma cells on THP1 monocytes or THP1 differentiated macrophages.

## 2. Materials and Methods

### 2.1. Human Subjects

Human studies were carried out in the Sleep Clinic of the Hospital Universitario Miguel Servet, a large teaching university hospital in Zaragoza, Spain, as part of the EPIOSA study (NCT02131610) [35] and in a multicenter trial conducted by the Spanish Sleep Network to assess the efficacy of CPAP in women with OSA (NCT02047071; [36,37]. All studies were approved by the appropriate human subject bioethics committees and all participants provided signed informed consents. Adult patients (mean age: 48.5 ± 6.7 years; male:female: 1:1; range: 20–65 years) with polysomnographically or polygraphically diagnosed OSA at baseline (V1) and after 12 months of adherent CPAP treatment (6.0 ± 2.1 h/night throughout the duration) (V2) were selected into this study (*n* = 26). Data from all sleep studies were scored using AASM guidelines [38] by trained personnel that were blinded to the aims or nature of the current study. Optimal titration of CPAP was implemented by using auto-CPAP (Autoset-T; ResMed, Sydney, Australia), according to the guidelines of the Spanish Sleep and Breathing Group [39]. 

Fasting blood samples were drawn at V1 and V2 using a 21G butterfly needle into ethylenediaminetetraacetic acid (EDTA) (PreAnalytix GmbH, Bene, Hombrechtikon, Switzerland). Plasma was separated by centrifugation and stored at −80 °C until analyses. A detailed description of Materials and Methods is provided in the Supplementary Materials. These data include exosome isolation, characterization from plasma and cell culture conditions, in vitro proliferation and migration, Western blotting, semi-quantitative reverse transcription–PCR (qRT-PCR), and miRNA isolation from exosomes derived melanoma cells. 

### 2.2. Cell Lines and Culture Reagents

Three human primary melanoma cells, namely CRL-1424, CRL-1619 and CRL-1675 were purchased from the American Type Culture Collection (ATCC, Manassas, VA, USA). Human skin malignant melanoma derived from adult male (31 years old) were grown in McCoy’s 5a Medium Modified (CRL-1424, Cat. # 302007). Human skin malignant melanoma derived from adult female (54 years old) were grown in Dulbecco’s Modified Eagle’s Medium (CRL-1619, Ca. # 302002). Human malignant melanoma cells derived from metastatic site of subcutaneous tissue derived from an adult male (29 years old) were grown in McCoy’s 5a Medium Modified (HTB-63, Cat. # 302007). The three cell lines were selected based on the fact that they have different known gene mutations: CRL-1424 is mutant in BRAF (+/+), CDKN2A (+/+), and STK11 (+/+); CRL-1619 is mutant in BRAF (+/−), CDKN2A (+/+); CRL-1675 cell is mutant in CDKN2A (+/+), not STK11, but also mutant in PTEN (+/+). Cells and culture media were obtained from ATCC (Manassas, VA, USA). Cells were grown in the recommended medium supplemented with 10% fetal bovine serum (Gibco, Gaithersburg, MD, USA), and an antibiotic/antimycotic solution at final concentrations of 100 U/mL penicillin, 100 μg/mL streptomycin (Sigma-Aldrich, St. Louis, MO, USA). Cells were maintained in T-25 tissue culture flasks (Techno Plastic Products AG, Trasadingen, Switzerland) in a standard humidified incubator with 5% CO_2_ balanced-air at 37 °C.

### 2.3. Exosome Isolation

#### 2.3.1. Plasma Exosomes

Exosomes were isolated from plasma samples using the Total Exosome Isolation Kit (TEIR) according to the manufacturer’s protocol instructions (Life Technologies, Carlsbad, CA, USA). After isolation, exosomes were carefully characterized as previously reported [32,40,41,42] based on approaches that strictly adhere to MISEV2018 guidelines [43]. Briefly, plasma was centrifuged at 2000× *g* for 20 min to remove cell/debris. The supernatants were collected. Plasma was then pre-filtered using 0.22 μm centrifuge tube filter (Sigma-Aldrich, St. Louis, MO, USA), and for 120 μl of plasma, 60 μl 1× PBS was added followed by 6 μl of proteinase K (Life Technologies, Carlsbad, CA, USA). After incubation at 37 °C for 12 min, 36 μL of TEIR buffer was added and incubated immediately at 4 °C for 35 min. This was followed by centrifugation at 10,000× *g* at 22 °C for 6 min, and pellets were solubilized in 60 μL of 1× PBS. Plasma samples were filtered again using 0.22 μm centrifuge tube filter (Sigma-Aldrich, St. Louis, MO, USA) to remove any large molecules. Exosomes samples were stored at −20 °C until use.

#### 2.3.2. Cell Culture Exosomes

Exosomes derived from melanoma cell supernatants after exposures to normoxia or IH were isolated using TEIR as per the manufacturer’s protocol. Briefly, supernatants were centrifuged at 2000× *g* for 20 min followed by filtration using 0.22 µM filter. The reagent (500 µL) was added to 1000 µL ml of cell media sample, and the solution was incubated overnight at 4 °C. The precipitated exosomes were recovered by standard centrifugation at 10,000 × *g* for 75 min, and the pellet was then re-suspended in PBS and stored at −20 °C.

### 2.4. Exosome Uptake by Melanoma Cells

Human plasma exosomes were labeled with the fluorescent linker PKH67-Green (Sigma-Aldrich, St. Louis, MO, USA). Second, exosomes derived from human melanoma cells exposed to either IH or normoxia for 48 h were labeled with fluorescent linker PKH26-Red (Sigma-Aldrich, St. Louis, MO, USA). For details about IH exposures, please see IH section below. Labeled exosomes were further incubated at 37 °C for 10 min. Samples were filtered through a microspin column G-25 (Sigma-Aldrich, St. Louis, MO, USA) to remove unbound dye. The reaction of all samples was stopped by adding ExoQuick-TC reagent (System Biosciences, Palo Alto, CA, USA), followed by placing the labeled exosome samples at 4 °C for 40 min following by centrifugation for 3 min at 14,500× *g*. The pellets were suspended in 1× PBS, and the labeled exosomes were added to human melanoma cells for 24 h in a cell culture incubator at 37 °C. The cell membranes were permeabilized by incubation with 0.25% (*v*/*v*) Triton-X-100 in PBS for 10 min. The labeled exosomes were monitored for delivery into the intended cells using a Leica SP5 Tandem Scanner Spectral 2-photon confocal microscope (Leica Microsystems, Buffalo Grove, IL, USA) with a 63× oil-immersion lens. As negative control, PKH67-Green was prepared and added to each cell plate with all reagents, but without any exosomes, to monitor background unincorporated dyes. Cell nuclei were imaged by staining with DAPI at a concentration of 1 μg/mL in PBS (Life Technologies, Carlsbad, CA, USA) at room temperature for 5 min [44,45,46].

### 2.5. Exosome Size Determination and Quantification

Morphological evaluation of exosomes was performed using electron microscopy as previously described [32,40]. Exosomes derived from patient plasma or melanoma cell cultures’ sizes distribution were assessed using electron microscopy (model Tecnai F30 at 300 KV; Thermo-Fisher, Hillsboro, OR, USA). Exosomes were placed on Formvar-carbon coated electron microscopy grids and allowed to stand for 5–10 min for exosome adsorption. Grids with adherent exosomes were washed three times with 25 μL drops of DPBS, then were fixed with 2% paraformaldehyde in DPBS for 7 min, followed by incubation with 25 μL drops of 2% uranyl acetate and then examined by electron microscopy. Size distribution of exosomes was inspected and quantified as previously described [32].

Exosomes, either from plasma or cell cultures, were quantified using either enzymatic fluorescent assays (FluoroCet #FCET96A quantitation kit; System Biosciences, Mountain View, CA, USA) according to the manufacturer’s protocol, or with the Spectradyne nCS1 nanoparticle analyzer (Spectradyne, Cherry Hills, CA, USA) which employs resistive pulse sensing technology, and further analyzed for selective subpopulation surface markers using FACS analysis (FACSCalibur, BD Biosciences), whereby purified exosomes were incubated with magnetic beads 9.1 μm with exosomes markers under gentle agitation at 4 °C overnight according to manufacturer’s recommendations. Control experiments were also performed with all the reagents and beads, but in the absence of exosomes. Twenty-five thousand events were computed, and data were analyzed using FlowJo Software (Tree Star, Inc, Ashland, OR, USA).

### 2.6. Proliferation and Migration Assays

All three human melanoma cell lines were used for experiments with OSA-derived plasma exosomes. Cells (2 × 104) were grown as described above, and a similar number of exosomes derived from either OSA-V1 or OSA-V2 were added for 24 h. Medium was removed from the plates 24 h after adding the exosomes, and the monolayers were rinsed with cold PBS and 200 μL of CyQUANT GR dye/cell lysis buffer (included in the CyQUANT kit, Invitrogen, Eugene, OR, USA) was added to each well. Fluorescence was measured using a microplate reader. The excitation maximum was 485 nm, and the emission maximum was 530 nm.

Migration assays were performed using human melanoma cells in 24-well trans-well inserts with 8 μm pore size (Corning, NY, USA). Cells (3.0 × 104) were cultured in the top in serum free medium in single culture, with and without exosomes isolated from OSA-V1 or OSA-V2 conditions. The cells were allowed to migrate for 40 h from the starved medium side to the lower compartment while using 10% FBS as chemoattractant. Cells were stained with 0.2% crystal violet solution (Sigma-Aldrich, St. Louis, MO, USA) and phase contrast images of the migrated cells were imaged using NIS-Elements AR vs. 3.10 imaging software. Sections were visualized with a Nikon Elipse Ti microscope using 4× objectives and photographed with a Cool SNAP EZ camera. The migration rate was determined by counting the number of cells contained in the photos of 5 random-selected different fields at original magnification by operators who were blinded to the experimental conditions. 

### 2.7. p-AMPK and p-AMPK Activator Western Blots

Human melanoma cells (CRL-1424) were grown as described above in 12-well plates in 10% FBS for 24 hr. Culture media were then replaced with 10% depleted FBS, and the cells were used under two different conditions. First, cells were treated with exosomes derived from OSA-V1, OSA-V2 (equal number of exosomes) or without exosomes for 24 h. Second, cells were treated with 15 µM of p-AMPK activator (PF06409577, Sigma-Aldrich (St-Louis, MO, USA) for 4 h, and equal numbers of exosomes were added to each individual well-plate for 24 h. Protein cell lysed concentrations were quantified using the BCA kit (Life Technologies, Rand Island, NY, USA). Equal amounts of total protein were electrophoresed using sodium dodecyl sulfate-polyacrylamide gel electrophoresis, SDS-PAGE, [10% and transferred into nitrocellulose membrane (Millipore, Billerica, MA, USA)]. The membranes were blotted with 5% skimmed milk and subsequently probed overnight at 4 °C with primary antibodies. The membranes were incubated with polyclonal p-AMPK ((Phospho-AMPKα (Thr172) (40H9), Cell Signaling, Danvers, MA, USA)) and AMPK polyclonal antibody (AMPKα, D5A2, Cell Signaling, Danvers, MA, USA) overnight at 4 °C, then washed three times for 10 min each with 25 mM Tris-HCL, pH 7.4, 3.0 mM KCl, 140 mM NaCl, and 0.05% Tween-20 (TBST), incubated with anti-rabbit immunoglobulin G:HRP-linked antibody (Cell Signaling Technology; 1:2000 dilution in blocking buffer with gentle agitation for 1 h at room temperature), and finally immunoreactive bands were visualized using an enhanced chemiluminescence detection system (Chemidoc XRS+; Bio-Rad, Hercules, CA, USA), and quantified by the Image Lab software (Bio-Rad, Hercules, CA, USA). The intensity of p-AMPK was adjusted by using total AMPK as a control. All the whole western blot figures can be found in the supplementary materials.

### 2.8. Cell Cultures and Intermittent Hypoxia

Melanoma CRL-1424 cells or CRL-1675 cells were grown in T25 flasks in recommended media as described above, supplemented with 10% fetal bovine serum (Gibco, Gaithersburg, MD, USA), and an antibiotic/antimycotic solution at final concentrations of 100 U/mL penicillin, 100 μg/mL streptomycin (Sigma-Aldrich, St. Louis, MO, USA) for 24 h. Subsequently, cell media were replaced with 10% depleted FBS (System Biosciences, LLC, Palo Alto, CA, USA) and cells were exposed to normoxia or IH for 48 h. Cells were placed in a cell culture incubator at 37 °C and 100% humidity, and connected to a gas source providing a gas mixture with either 21% O_2_ (normoxia) or 1% O_2_ (hypoxia) for 48 h alternating every 30 min. This profile generated nadir partial pressure of oxygen in the 3–7 mmHg range [47]. Cell culture supernatants were collected, and exosomes were isolated as described above.

### 2.9. qRT-PCR

Total RNA was extracted using RNeasy Kit (Qiagen, MD, USA) following the manufacturer’s instructions. Quantitative polymerase chain reaction analysis (qRT-PCR) was performed for selected messenger RNAs (mRNAs) using ABI PRISM 7500 System (Applied Biosystems, Foster City, CA, USA). For RNA, cDNA was synthesized using 200 ng of total RNA using a High-Capacity cDNA Archive Kit (Applied Biosystems, Foster City, CA, USA). The thermal cycling conditions were 95 °C for 10 min, followed by 40 cycles at 94 °C for 15 s, 60 °C for 30 s, and 72 °C for 30 s. Human 18S gene was used as a reference gene to normalize the expression ratios. The cycle number (Ct) values were averaged and the difference between the housekeeping gene Ct and the gene of interest Ct was calculated to determine the relative expression of the gene of interest using the 2^−ΔΔCt^ method.

### 2.10. Exosomal miRNA Isolation from Melanoma Cells

Two different melanoma cells CRL-1424 and CRL-1675 were used and grown at 37 °C for 48 h supplement with 10% depleted FBS. Supernatants were collected and centrifuged at 2000× *g* for 35 min. Exosomes were isolated using TEIR for cell culture media (Life Technologies, Grand Island, NY, USA) as described by the manufacture protocol. The supernatant was transferred into a new centrifuge tube and 500 µL of TEIR was added for 1 mL of cell culture media, and further incubated at 4 °C overnight for 16 h. Following incubation, the samples were centrifuged at 10,000× *g* for 75 min at 4 °C. The supernatants were discarded, and the pellet was re-suspended in 1X PBS.

#### 2.10.1. miRNAs Microarray Processing and Analysis

Total RNA including miRNA was isolated from purified exosomes obtained from cells either exposed to normoxia or IH for 48 h using miRNeasy Mini Kit-column-based system (Cat. # 217004, Qiagen, Germantown, MD, USA) following the manufacturer’s instructions (Qiagen, Turnberry Lane, Valencia, CA, USA) [48]. Exosomes were homogenized in QIAzol Lysis Reagent. After addition of chloroform, the homogenate was separated into aqueous and organic phases by centrifugation using the miRNeasy Mini Kit (Qiagen^®^) which combines phenol/guanidine-based lysis of samples and a silica membrane column-based purification as described by the manufacturer protocol [47]. The RNA quality and integrity were determined using the Eukaryote Total RNA Nano 6000 LabChip assay (Agilent Technologies, Santa Clara, CA, USA) on the Agilent 2100 Bioanalyzer [47].

Each sample was prepared according to Agilent’s miRNA recommended approach using the one-color technique and profiled on the Agilent human miRNA microarray (Agilent Technologies, Santa Clara, CA, USA) [47]. Each array consisted of 60-mer DNA probes synthesized in situ that represent 2006 human miRNAs. The labeled RNAs were hybridized to custom human miRNAs microarrays 8 × 60 K (Agilent Technologies, Santa Clara, CA, USA). Following hybridization and washing, the arrays were scanned with an Agilent microarray scanner using high dynamic-range settings as specified by the manufacturer. Microarray results were extracted using Agilent Feature Extraction software (v12.0) to quantify signal intensities [32,47]. The quality control for each miRNA was evaluated based on their Agilent Feature Extraction quality control report. miRNA microarray data were log-base 2 transformed and quantile normalized. The normalized data were expressed as the difference of log of g processed signal (Agilent Feature Extraction). Undetected probes were excluded from further analysis. Background-subtracted intensities were normalized for detected miRNA probes using the quantile method across all miRNA microarray experiments as described previously.

#### 2.10.2. Target Gene Prediction and Function Enrichment Analysis

To identify the underlying target genes of DE miRNAs, the miRWalk2.0 database (zmf.umm.uni-heidelberg.de/apps/zmf/mirwalk2/ accessed on 16 July 2021) was used. This database contained a total of 12 bioinformatic algorithms, including DIANA microT (version 4.0), DIANA microT CDS (version 5.0), miRanda rel (version 1.0), mirBridge (version 1.0), miRDB (version 4.0), miRmap (version 1.0), miRNAMap (version 1.0), PicTar (version 2.0), PITA (version 6.0), RNA22 (version 2.0), RNAhybrid (version 2.1) and Targetscan (version 6.2). The Database for Annotation, Visualization and Integrated Discovery (DAVID) online tool (version 6.8; http://david.abcc.ncifcrf.gov, accessed on 16 July 2021) was used for Gene Ontology (GO) term and Kyoto Encyclopedia of Genes and Genomes (KEGG) pathway enrichment analyses of all target genes of DE miRNAs. A *p* value of <0.05 was set as the cut off value. 

### 2.11. THP-1 Monocytes and Differentiation to Macrophages

THP-1 cells were used in two different conditions. First, THP-1 monocytes (0.2 × 106) cells were seeded in 12-well plates for 48 h. Second, THP-1 (0.2 × 106) cells were differentiated into macrophages in 12-well plates containing 2 mL of the RPMI 1640 medium containing 150 µM phorbol 12-myristate 13-acetate (PMA) for 48 h at 5% CO_2_ at 37 °C. Media were replaced with depleted FBS and exosomes derived from IH or normoxia were added for 24 h. Cells were collected and used for either flow cytometry to evaluate cell polarity using specific antibodies or for total RNA isolation to evaluate macrophage-related gene expression. 

For immunofluorescence analysis, cells were stained with different antibodies such as APC/Cy7 anti-human CD11b (Cat. # 301341), antibody. Cells were stained with 1µg antibody in 100 µL. Cell Staining Buffer was then added and further incubated at 4 °C for 20 min in the dark. Samples were washed twice with Cell Staining Buffer and pellets were resuspended in 0.5 mL of Cell Staining Buffer. Flow cytometric measurements were performed using a 4-color FACS Calibur (Becton Dickinson, San Jose, CA, USA). Following incubation, samples were washed and resuspended in PBS and 10,000 events were recorded. 

### 2.12. Data Analyses

Results are presented as means ± SD, unless stated otherwise. Comparisons of all quantitative data groups were performed using unpaired Student t-tests, non-parametric tests, or ANOVA procedures as appropriate depending on the variable distributions. Statistical analyses were performed using SPSS version 21.0 (SPPS Inc., Chicago, IL, USA). For all comparisons, a two-tailed *p* < 0.05 was considered to define statistical significance.

## 3. Results

### 3.1. Human Subjects

**Figure 1:**
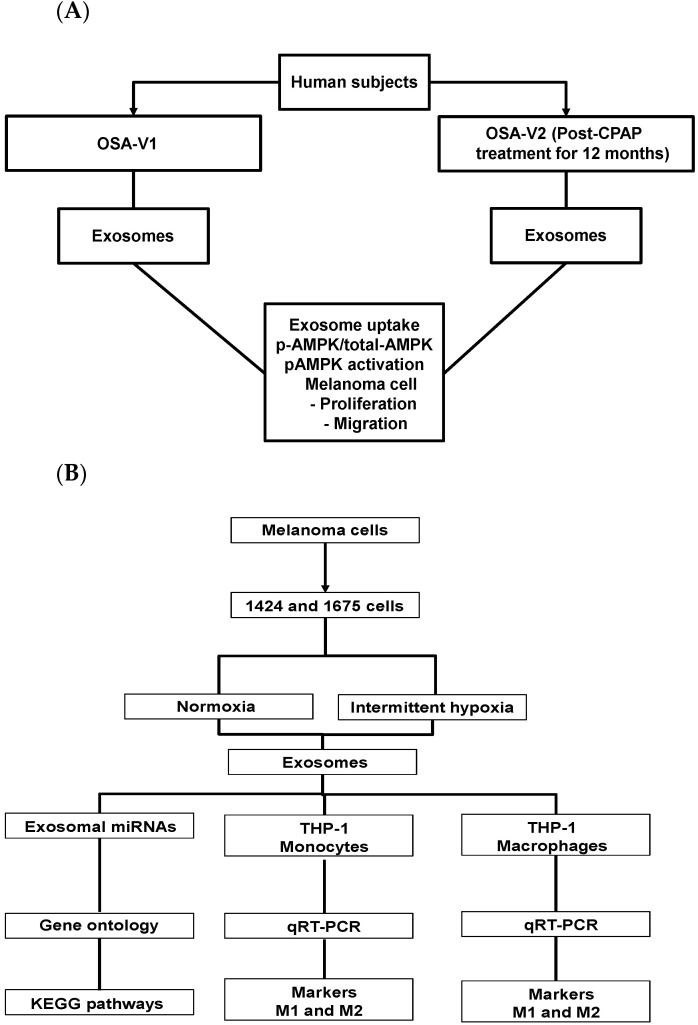
Schema illustrating the experimental design and data analyses. (**A**) Plasma exosomes were isolated and purified from obstructive sleep apnea (OSA) patients either before CPAP treatment (OSA-V1) or after treatment (OSA-V2). (**B**) Diagram illustrating cell culture exposed to normoxia and IH for 48 h, and exosomes characterization, exosomal miRNAs microarrays profiling, Gene Ontology and KEGG pathways.

Subjects suffering from moderate to severe OSA were assessed at two different times: (A) untreated (OSA-V1; *n* = 26; 20 males), and (B) treated with CPAP for a period of 12 months (OSA-V2, *n* = 26), as shown in Figure 1A. There were no significant differences in subject BMI between the V1 and V2 conditions (31.7 ± 3.7 kg/m^2^). Twelve of the subjects were hypertensive and 16 reported excessive daytime sleepiness (Epworth scores > 12).

However, the apnea–hypopnea index (AHI), a measure of OSA severity was markedly higher among OSA-V1 subjects (70.13 ± 16.78 events/h) compared to OSA-V2 (2.71 ± 1.32 events/h; *p* = 0.0001). In addition, significant differences emerged in diastolic blood pressure between OSA-V1 (82.31 ± 8.59 mmHg) and OSA-V2 (73.15 ± 10.12 mmHg; *p* = 0.01). In parallel, three commercially available melanoma cell lines (CRL-1424, CRL-1619, and CRL-1675) were exposed to IH or normoxia as indicated in Figure 1B.

### 3.2. Exosome Characterization and Uptake

Exosomes derived from OSA subjects or from the supernatants of the cell lines were characterized as mentioned. Exosome counts were determined in OSA-V1 (4.53 × 10^8^/mL) or OSA-V2 (4.48 × 10^8^/mL; *p* = 0.24, *n* = 20/group). The purity of the plasma-derived exosomes was also confirmed by TEM (Figure S1A). Flow cytometry analyses for exosome surface markers including tetraspanin (CD63) in the presence or absence of exosomes were performed as shown in Figure S1B. In plasma exosomes labeled with PKH67, we observed exosome internalization in melanoma cells as fluorescent membrane dye (Figure S2), illustrating that exosomes were functionally intact. Similar procedures were applied for exosomes derived from melanoma cell cultures, including Spectradyne nCS1, flow cytometry (CD63), and TEM (University of Missouri Microscopy Core, Columbia, MO, USA) as described in Methods. Exosome counts in CRL-1424 cell culture supernatants exposed to IH (3.46 ± 0.23 × 10^8^/mL) or normoxia (3.46 ± 0.22 × 10^8^/mL; *n* = 8/group; *p* = 0.16), and in CRL-1675 cell culture supernatants (3.31 ± 0.21 × 10^8^/mL in IH; 3.18 ± 0.19 × 10^8^/mL in normoxia; *n* = 8/group; *p* = 0.23) were similar and unaltered by the type of exposure.

### 3.3. Cell Proliferation and Migration

**Figure 2:**
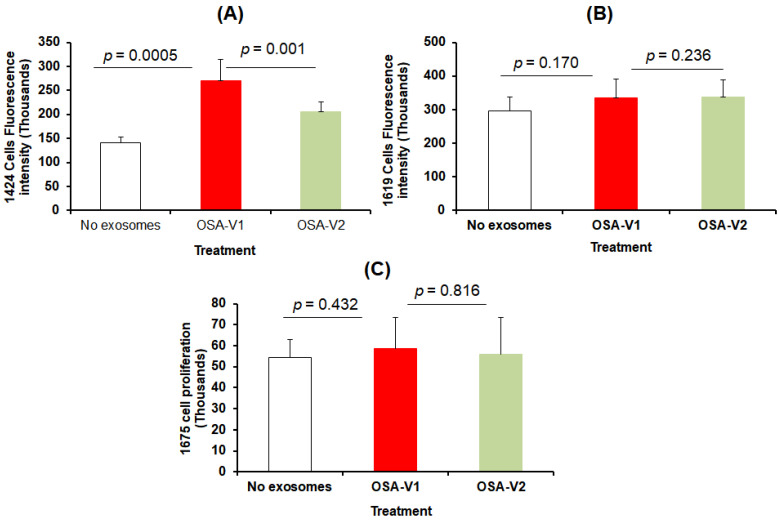
Effects of plasma exosomes-derived from OSA patients before CPAP treatment (OSA-V1) and after adherent CPAP treatment for 1 year (OSA-V2) on melanoma cells proliferation. Exosomes-derived from OSA patients were incubated in 96-wells plates for 24 h. The cells were treated with CyQUANT GR dye/cell lysis buffer, and the fluorescence was measured using a microplate reader (the excitation maximum was 485 nm, and the emission maximum was 530 nm). (**A**) represents the average of fluorescence intensity of cell proliferation for CRL-1424 cells, (**B**) CRL-1619 cells, and (**C**) CRL-1675 cells with statistical significance set at *p* < 0.05 (*n* = 20/condition).

The effect on proliferation and migration of plasma-derived exosomes on melanoma cells was assessed in CRL-1424, CRL-1619, and CRL-1675 cell lines. As shown in Figure 2, exosomes derived from OSA-V1 increased proliferation of CRL-1424 cells when compared to OSA-V2 (average of fluorescence signal OSA-V1: 270,303.88 ± 44,237.86 ×10^3^ vs. OSA-V2: 204,727.75 ± 21,704.25 × 10^3^, *p* = 0.001, *n* = 12; Figure 2A); however, no significant differences in proliferation emerged for either CRL-1619 or CRL-1675 cell lines after treatment with exosomes (Figure 2B,C).

**Figure 3:**
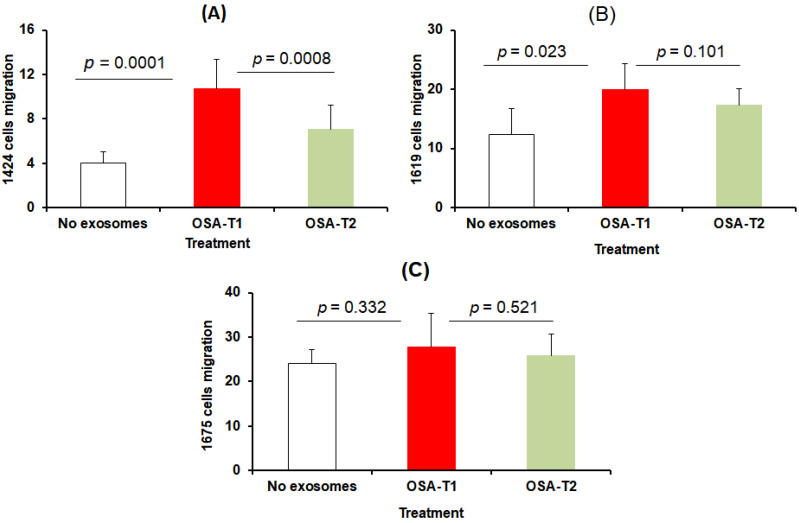
Effects of plasma exosomes-derived from OSA patients before CPAP treatment (OSA-V1) and after adherent CPAP treatment for 1 year (OSA-V2) on melanoma cell migration. Cells were seeded in 24-well trans-well inserts with 8 μm pore size. Cells were allowed to migrate for 40 h, and then were stained with 0.2% crystal violet solution followed by phase contrast imaging. The migration rate was determined by counting the number of cells contained in the photos of 5 different fields at original magnification. (**A**) represents average of different fields for each subject for each group using CRL-1424 cells, (**B**) CRL-1629, and (**C**) CRL-1675, (*n* = 20/group), with statistical significance set at *p* < 0.05.

In migration assays, the three melanoma cell types were seeded in trans-wells individually and treated with exosomes derived from OSA-V1 and OSA-V2, cells were then counted (Figure 3A–C). We found that only CRL-1424 cells showed a significant increase in migration when cells were treated with exosomes from OSA-V1 (1000.74 ± 112.62) compared to the same patients treated with CPAP OSA-V2 (700.10 ± 103.18; *p* = 0.008; *n* = 20) (Figure 3A). However, there were no significant differences in melanoma cell migration in either CRL-1619 or CRL-1675 cells after exposure to exosomes from OSA-V1 compared to OSA-V2, as shown in Figure 3B,C.

### 3.4. Effect of Exosomes on AMPK 

**Figure 4:**
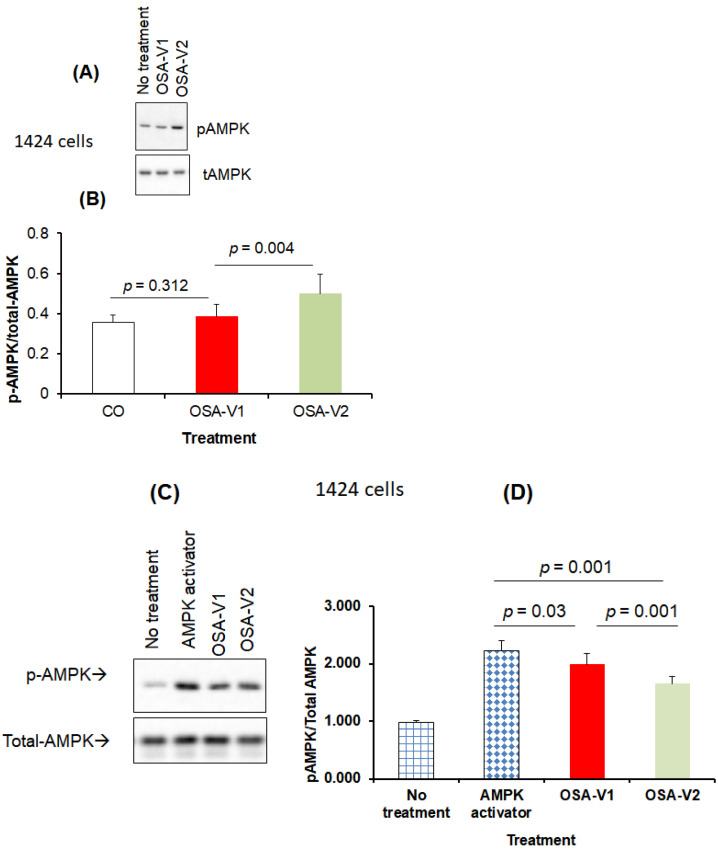
Effects of plasma exosomes-derived from OSA patients before CPAP treatment (OSA-V1) and after adherent CPAP treatment for 1 year (OSA-V2) on the presence or absence of AMPK activation using naïve melanoma cells (CRL-1424). Cells were grown on 12-well plates and treated with exosomes for 24 h in the presence or absence of AMPK activator. In the presence of AMPK activator, cells were also grown in 12-well plates and treated with AMPK activator for 4 h, and media were then removed and replaced with new media followed by adding exosomes for 24 h. Panel (**A**) shows a representative image of Western blots for no exosomes, OSA-V1 and OSA-V2 on CRL-1424 cells. Panel (**B**) shows a histogram for the densitometric quantification of Western blots bands (*n* = 8/condition) with statistical significance set at *p* < 0.05. Panel (**C**) shows a representative Western blot for CRL-1424 cells (no exosomes, OSA-V1 and OSA-V2). Panel (**D**) shows a histogram for the densitometric quantification of Western blots bands (*n* = 8/condition) with significance set at *p* < 0.05.

AMPK is known to be activated under metabolically stressed conditions by turning on energy-generating catabolic processes such as fatty acid oxidation and glycolysis, while mitigating energy-consuming anabolic pathways, such as carbohydrate, lipid, and protein biosynthesis. We therefore examined the effects of OSA-derived plasma exosomes on phosphorylation of AMPK in naïve CRL-1424 and CRL-1675 melanoma cells (Figure 4, Panel A). In CRL-1424 cells, OSA-V1 exosomes induced significant decreases in pAMPK/tAMPK ratios (0.38 ± 0.06) compared to OSA-V2 (0.52 ± 0.09; *p* = 0.004, *n* = 8; Figure 4, Panel B), while no differences emerged for CRL-1675 (data not shown). Next, CRL-1424 cells were treated with an AMPK activator for 4 h, and then treated with exosomes from either OSA-V1 or OSA-V2 (Figure 4, Panel A). The ratio of pAMPK/tAMPK was markedly increased by the AMPK activator (2.23 ± 0.17) (Figure 4, Panel B). The addition of AMPK activator and exosomes derived from OSA-V1 resulted in higher pAMPK/tAMPK (1.98 ± 0.21) compared to exosomes from OSA-V2 (1.66 ± 0.16; *p* = 0.001, *n* = 8; Figure 4, Panel B), but not to the same level as AMPK activator alone, suggesting that exosomes in plasma of OSA patients may regulate AMPK in cancer cells susceptible to IH. 

### 3.5. Effect of Intermittent Hypoxia on Exosomes Released by Melanoma Cells

**Figure 5:**
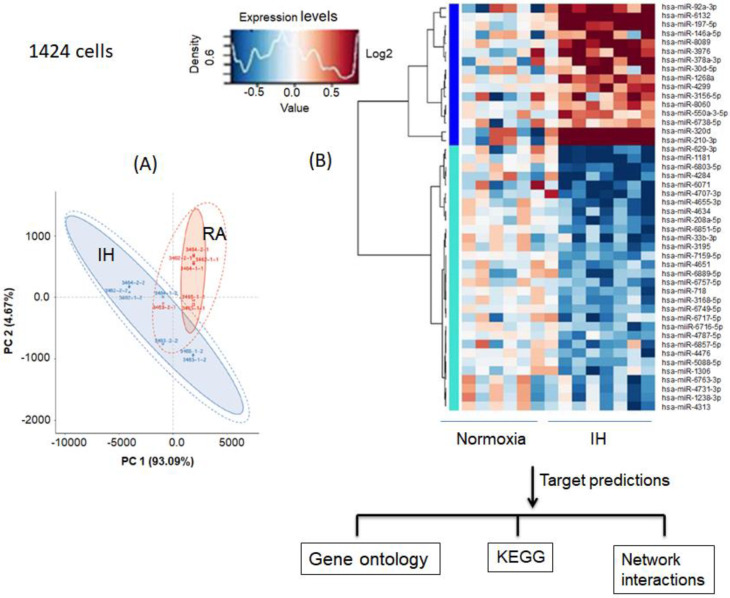

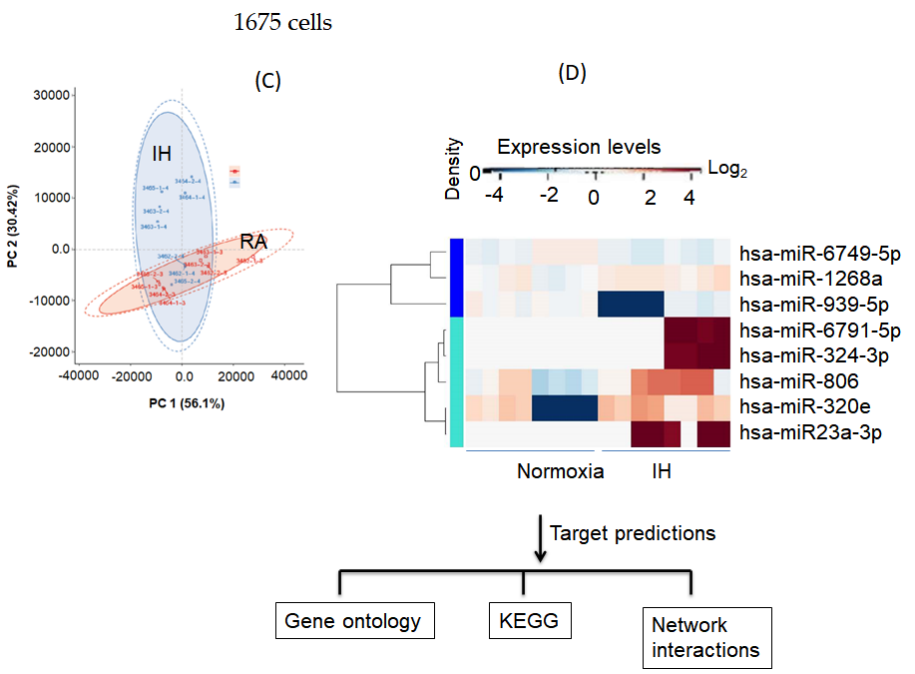
Differentially expressed exosomal miRNAs derived from the supernatants of melanoma cells (CRL-1424 and CRL-1675) after exposures to normoxia (RA) and IH conditions. Panel (**A**) shows a principal component analysis (PCA) plot of exosomal miRNAs for CRL-1424 cells. PCA clustering of miRNA expression by subject group is denoted by different colors and reveals complete and accurate separation of miRNA expression across the subjects from the two groups. Panel (**B**) shows a heatmap illustrating miRNA expression patterns (dark red, increased miRNA expression, light blue, reduced miRNA expression in exosomes. Panel (**C**) shows the PCA of exosomal-miRNA. Panel (**D**) shows a heatmap illustrating miRNA expression patterns for CRL-1675 cells. The dendrograms show hierarchical clustering representing the similarities and dissimilarities in expression profiles among individuals and miRNAs. In silico target gene predictions were used for GO, KEGG pathways, and gene network interactions.

We identified unique exosomal miRNAs for melanoma cell cultures (CRL-1424 and CRL-1675) exposed to 48 hrs of IH when compared to normoxic conditions (Figure 5). Principal component analysis (PCA), and heat-map clustering revealed consistent group separation and categorical assignments, which were concordant with the experimental groups, and showed substantial differences in miRNA expression profiles for exosomes from CRL-1424 (Figure 5, Panels A–D) and CRL-1675 (Figure 5, Panels A and B), respectively. The overall magnitude of the changes in exosomal miRNA cargo with IH was substantially more robust in CRL-1424 than in CRL-1625. Indeed, we found 46 miRNA that were differentially expressed in exosomes from IH-exposed CRL-1424 cells when compared to normoxic conditions, while only 8 miRNAs were differentially expressed in exosomes from CRL-1675 cells following IH exposures (Figure 5).

### 3.6. Melanoma Gene Ontology and KEGG Pathways

We next examined the predicted gene targets of the differentially expressed miRNAs in exosomes released after exposures to IH from CRL-1424 and CRL-1675 cells, and based on several prediction tools we identified 8172 gene targets for CRL-1424 and 2073 gene targets for CRL-1625, respectively. To assess the potential biological roles of these exosomal miRNA target genes, we performed GO classification enrichment analysis. For CRL-1424, there were 48 differentially enriched terms involved in biological processes, 42 in the cellular component, and 22 in the molecular functions (Table S1A–C), respectively, while for CRL-1675 there were 19 involved in biological processes, 8 in cellular component, and 5 in molecular functions (Table S2A–C). 

In the KEGG database, we identified several pathways that were differentially and putatively targeted by exosomes from IH-exposed CRL-1424 cells including Ras signaling pathway (hsa04713, *p* =1.72 × 10^−7^), AMPK signaling pathway (hsa04010, *p* = 5.40 × 10^−6^), and cAMP signaling pathway (hsa04261, *p* =5.99 × 10^−5^), and MAPK signaling pathway (hsa04550, *p* =6.44 × 10^−6^) (Table 1). Conversely, in the exosomes of IH-exposed CRL-1675 cells, the miRNAs that were differentially expressed were likely to target pathways such as transcriptional mis-regulation in cancer (hsa05202, *p* = 2.37 × 10^−4^), the ErbB signaling pathway (hsa04012, *p* = 4.87 × 10^−4^) and the Sphingolipid signaling pathway (hsa04071, *p* = 9.34 × 10^−4^) (Table 2).

**Figure 6:**
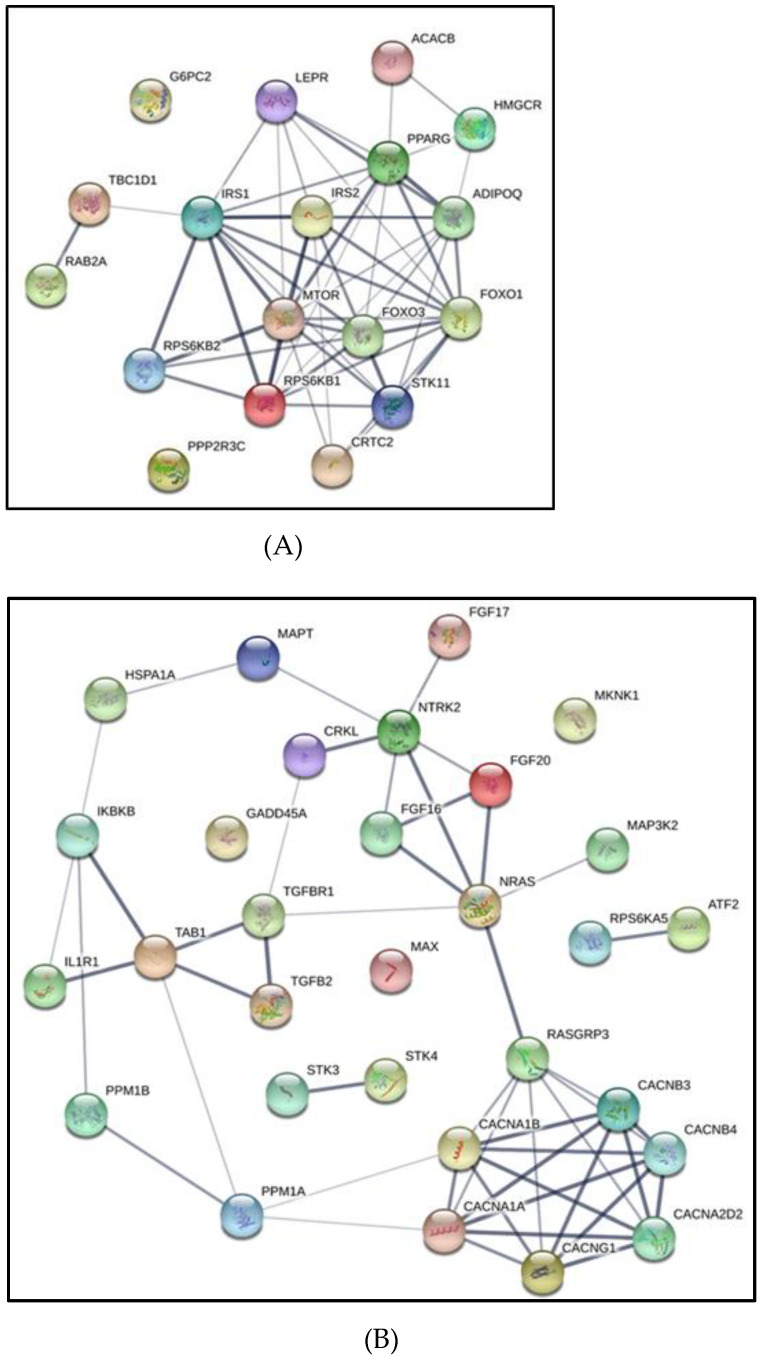

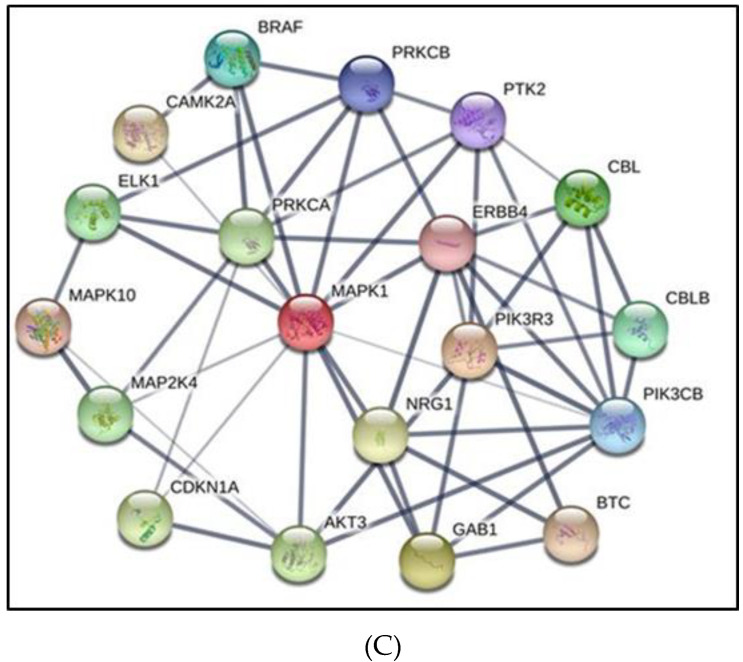
Gene network representation of interactions between gene target predictions of exosomal-miRNAs derived from melanoma cells (CRL-1424) exposed to normoxia or IH conditions. Three gene networks were generated from gene lists presented in Table 1. Panel (**A**) represents the gene network for the AMPK pathway, Panel (**B**) MAPK pathway, and Panel (**C**) cAMP pathway.

In addition, using String software we constructed regulatory gene networks for the predicted gene targets, and in light of the differences in exosome effects on cell proliferation and migration focused on genes involved in AMPK network (Figure 6A), MAPK (Figure 6B), and cAMP network (Figure 6C), respectively. As shown in Table 1 several pathways were highlighted including Ras signaling, PI3K-Akt signaling, AMPK signaling, MAPK signaling, and cAMP signaling in CLR-1424 cells.

### 3.7. Effects of Exosomes Derived from Melanoma Cells on Naïve THP1 Cells

Since monocytes and macrophages play a key role in innate immune surveillance and in tumor biology, we examined the polarity effects of exosomes derived from melanoma cells exposed to either IH or normoxia on naïve THP1 monocytes and macrophages using specific genes for M1 markers (namely *CCR7*, *CXCL10* and *IL6*), and for M2 markers (*CD206*, *CD163*, and IL10). We used flow cytometry for CD11b surface marker to confirm the induction of THP1 monocytes into macrophages (Figure S3; *n* = 6). As shown in Table 3 and Table 4, exosomes derived from CRL-1424 and CRL-1675 exposed to IH increased the expression of *CXCL10* and *IL6* in THP1 monocytes when compared to exosomes from the same cells under normoxic conditions (Table 3). Exosomes-derived from CRL-1675 exposed to IH increased the expression of *CCR7*, *CXCL10* and IL6 of THP1 macrophages, while only *CXCL10* expression was increased after exosomes from IH-exposed CRL-1424 cells (Table 4).

## 4. Discussion

This study shows that plasma exosomes derived from OSA patients induce divergent effects on the proliferation and migration of different melanoma cell lines when compared to exosomes from the same patients after receiving adherent treatment with CPAP for one year. Thus, heterogeneity of melanoma cell lines selected for their underlying mutations in specific genes reveals discrepant responses to the cargo of plasma exosomes obtained from patients with OSA. Similarly, the intrinsic exosomal cargo changes of two melanoma cell lines with divergent proliferative and migration responses to exosomes from OSA patients markedly differed and revealed differential miRNA cargos with distinct downstream gene targets and pathways. One of the pathways in melanoma cell lines, i.e., CRL-1424, implicated AMPK as being involved in the heterogeneous responses to exosomes, while no such evidence was present in the other melanoma cell line, namely CRL-1675. Experiments conducted to assess pAMPK/tAMPK ratio changes confirmed such assumptions. Furthermore, treating melanoma cells with a selective AMPK activator along with exosomes of OSA patients before and after treatment further corroborated the potentially important role of deregulated cell metabolism in CRL-1424 in the context of IH. Finally, exosomes derived from all three melanoma cell lines alter the expression of M1 and M2 makers in naïve monocytes or induced macrophages in vitro, albeit with slight differences across the cell lines, with such effects being more prominent following IH exposures.

OSA is a highly prevalent disease that has been estimated to affect nearly a billion people around the world, is associated with an increased risk for cardiovascular and metabolic diseases and overall mortality, and requires lifelong adherent treatment, usually with CPAP [49,50,51,52,53]. In recent years, significant associations between OSA and prevalence, incidence and mortality of several cancers have emerged, with melanoma being consistently and prominently represented [7,9,19,54]. Both the IH and recurrent arousals that constitute two of the major hallmark features of OSA have been shown to promote activation and propagation of intermediate mechanisms linking OSA to the increased cancer risk [55]. For example, mice exposed to IH and injected with cancer cells developed tumors that not only exhibited accelerated growth, but also displayed enhanced invasiveness toward adjacent tissues that were associated with enhanced abundance of TAMs [56]. Several studies have reported that exosomes are mediators of cancer aggressiveness and of metastatic processes, whereby exosomes derived from either normal or from cancer cells can promote angiogenesis, invasion, and proliferation in recipient cells to support tumor growth [57,58,59,60]. In this context, we have previously shown that plasma exosomes from patients with OSA foster tumor cell aggressiveness in vitro, but have also identified substantial variability of exosome-induced responses across different cancer types (data not shown). In the present study, we show for the first time that human melanoma cell lines exhibit markedly disparate response characteristics to plasma exosomes from patients with OSA, that they are also divergent in their intrinsic proliferative and migration responses to IH, and that the exosomes they release include different miRNA cargos.

Progression of melanoma is dependent on cross-talk between tumor cells and the adjacent microenvironment, the latter being enriched in exosomes constantly secreted by cancer cells and by many other cells, both neighboring and distantly located. For example, melanoma cell-derived exosomes can enhance the emergence and expansion of epithelial-mesenchymal transition in primary melanocytes through paracrine/autocrine signaling in the tumor microenvironment [61]. In this study, we found that exosomes derived from OSA-V1 increased proliferation and migration of naïve CRL-1424 melanoma cells compared to the exosomes of the same patients after one year of adherent OSA treatment. Such responses were not apparent in CRL-1675 cells, indicating that the underlying genetic mutations in each melanoma tumor may play an important role in the determination of the specific exosomal cargo elements underlying these processes. Since the unique differences in CRL-1424 melanoma cells reside in the presence of a STK11 mutation, a gene encoding for serine/threonine kinase family that acts not only as a tumor suppressor, but is also involved in metabolic regulation, it is possible that the effects of OSA-derived plasma exosomes on the CRL-1424 melanoma cells may specifically involve STK11-regulated pathways. Indeed, STK11 is a direct activator of AMPK, and as such, the presence of genetically deregulated STK11 seems to translate into reduced AMPK activation as our current findings indicate [62,63]. Targeted experiments aiming to determine the specific pathways underlying the differences in cellular responses between the various melanoma cell lines when treated with exosomes from OSA patients are clearly beyond the scope of the current study. However, initial attempts in this direction identified several differentially expressed miRNAs in exosomes from CRL-1424 exposed to IH when compared to normoxic conditions that were also not represented in the CRL-1675 melanoma cell line. Of the major known pathways regulated by these differentially expressed miRNAs, AMPK was prominently represented, and experiments revealed marked differences in the effects of OSA-derived exosomes on AMPK phosphorylation between CRL-1424 and CRL-1675. These results further reinforce the conceptual framework that heterogeneous responses of melanoma cells to either IH directly, or to exosomes from patients whose disease is characterized by IH involve discrepant regulation of metabolic genes and pathways in general, and more specifically of AMPK. It has been reported that AMPK is activated by metabolic stresses that reduce ATP production such as hypoxia [64]. Activation of AMPK directly restricts translational initiation and protein synthesis through inhibition of translation elongation factor 2 (EF2) [65]. Of note, AMPK is activated by the presence of high levels of AMP/ATP ratios or by reduced cellular energy status, both of which lead to decreased metabolic activity. Attenuated proliferation is also a direct consequence of AMPK activation in energy deficient cells [66]. Furthermore, cancer cells, as opposed to non-cancerous cells, are able to suppress the activation of AMPK despite the imbalance between cellular energy vs. rate of proliferation and thus sustain active growth and invasion [67].

Exosomes have indeed emerged as important players of cancer initiation and progression through cell–cell communication, with their uniquely regulated cargo potentially promoting transformation, growth and invasion of cancer cells under specific conditions such as in OSA. Additionally, exosomes present a novel mode of communication between malignant cells and monocytes/macrophages; however, the mechanisms behind this potential role for exosomes in melanoma are only partially understood [32,68]. Here, we found that exosomes from melanoma cells were selectively enriched in specific miRNAs that differed based on the specific mutations in the melanoma cell line (i.e., CRL-1424 and CRL-1675) and such differences in exosomal cargo were primarily apparent when melanoma cells were exposed to IH. However, subtle differences were detected between the effects of exosomes from the various melanoma cell lines exposed to IH on monocytes and macrophage polarity markers, suggesting that mutual interactions between the host immunity and the specific cancer cells may be modulated by the concurrent presence of IH, and that the nature of such modulation is dictated by the genotype-phenotype profile of the melanoma cell line.

It is now widely accepted that hypoxia enhances the malignant properties of tumors, and can strongly influence tumor growth, differentiation and migration [69,70]. Several studies have identified specific patterns of miRNA dysregulation in human cancers, including melanoma [61,71,72]. Furthermore, miRNAs have emerged as central players in cancer biology, and have been demonstrated their unique utility in determining tumor type, prognosis and response to therapy, being intimately involved in both the tumor cell-intrinsic properties and in the generation of the tumor microenvironment and in pro- and anti-oncogenic signaling [73]. Of further relevance to the present study, miRNAs are involved in the regulation of metabolic processes, specifically in response to hypoxic stimuli, and can mediate the metabolic switch from an oxidative to a glycolytic metabolic pathway [73]. To further delineate the effect of IH on melanoma cells, we isolated and examined their exosomal miRNAs profiles. First, to gain further insight into the cellular functions of melanoma exosomal miRNAs, GO and KEGG pathway enrichment analyses enabled the identification of predicted target genes among differentially expressed miRNAs within the exosomal cargo of each of the cells. As such, pathways such as PI3K/AKT, AMPK, and MAPK emerged as prominently regulated [73].

The MAPK and PI3K/AKT pathways contribute to the pathogenesis of melanoma. For example, mutations or loss of PTEN and dysregulation in expression of AKT, which positively regulates the G1/S phase progression in cell cycle, suppresses apoptosis and promotes cellular survival [74,75]. In addition, melanoma cell-derived exosomes can promote phenotype switching in primary melanocytes through paracrine/autocrine signaling and MAPK are involved in these processes [61,76,77,78].

Overall, we identified six miRNAs; three up-regulated (hsa-mir-146a, hsa-mir-30d, and hsa-mir-210-3p) and three down-regulated (hsa-mir-33b, hsa-mir-4787-5p, and 4731-3p), all of which have been implicated in some aspect of melanoma biological properties [73,79,80,81]. For example, miR-146a exerts a paradoxical role in melanoma tumor cells: this miRNA inhibits tumor metastatic behaviors through ITGAV inhibition, while it concurrently favors tumor growth by activating the AKT/PTEN pathway [82]. Similarly, miR-210 is among the now established hypoxia-induced miRNAs in melanoma, and has been shown to impair susceptibility to T-cell lysis by tumor cells [69,83]. It has been reported that miR-210 is a direct transcriptional target for the hypoxia-induced transcription factors, HIF1α and HIF2α [84]. Additionally, miR-210 plays a regulatory role in the inhibition of cell cycle arrest during hypoxic conditions, particularly by suppressing the MYC proto-oncogene antagonist, and by facilitating cell growth of tumor cells even when oxygen is not immediately available [85,86].

Among the down-regulated miRNAs, miR-33b was down-regulated in exosomes derived from CRL-1424 cells exposed to IH. These findings concur with previous studies showing that miR-33b is downregulated in highly metastatic breast cancer cell lines as well as breast cancer tissue samples, and that miR-33b expression is negatively correlated with clinical stage and lymph node metastasis of human breast cancer [80]. In addition, miR-30b/miR-30d is involved in the melanoma metastatic process and can act as immunosuppressive miRNAs [79]. The deranged expression of miR-30b/30d leads to the downregulation of GalNAc transferase GALNT7, resulting in suppression of immune cell activation and recruitment mediated by a high expression level of immunosuppressive cytokine IL-10 [79].

Monocytes play a pivotal role in host immunity, and upon activation, these cells can undergo differentiation into macrophages [87], the latter being key participants in tumor pathogenesis [88]. For example, M1 polarized macrophages possess anti-tumor functions whereas M2 tumor associated macrophage (TAMs) promote tumor growth [89]. Here, we used exosomes derived from three melanoma cells (CRL-1424, CRL-1619, and CRL-1675) which were exposed to IH or normoxia, and exposed naïve monocytes or macrophages to these exosomes to investigate their effects on M1 and M2 markers. These experiments provided initial insights into the differential effects of exosomes from different melanoma cell lines on monocyte/macrophage polarity. We should remark that while the M1 polarization induced by the exosomes from melanoma cells exposed to IH would predict a tumor-suppressive effect, we surmise that the host immune response-melanoma interactions may yield an opposite effect in vivo, further supporting the need for future exploration of functional responses in the context of tumor immunity.

Several limitations in the present study deserve mention. The number of OSA patients was relatively limited and merits expansion to a wider age range, OSA severity, and further exploration of potential differences across sexes. Although the effects of exosomal cargo of patients and melanoma cells lines were tested in vitro, their in vivo properties need to be examined, and the specific role of each miRNA of interest in the exosomal cargo needs to be mechanistically explored using agomir and antagomir strategies, both in vitro and in vivo. In addition, considering the fact that melanogenesis requires oxygen [90], assessment of this pathway in future studies may provide important insights as to underlying signal transduction elements involved in the observations reported herein. Despite these limitations, our study has several strengths, namely we show for the first time that exosomes from OSA patients promote proliferation and migration of melanoma cells in vitro, and that such effects are restricted to certain types of melanoma, indicating a remarkable specificity that may underlie unique therapeutic target opportunities. In addition, considering the differential effect of exosomes from OSA patients on the various cell lines, we identified AMPK-related pathways as being of critical importance in the interactions between OSA and melanoma. We also identified exosomal miRNA signatures corresponding to different mutations of melanoma cells when exposed to IH. These miRNAs may emerge as playing critical roles in tumor progression and metastasis. Finally, we showed that exosomes derived from melanoma cells differentially alter monocyte and macrophage phenotypes in vitro.

In summary, exosomes derived from OSA patients selectively enhance the proliferation and migration of melanoma cells in vitro, and such divergent effects appear to involve metabolic pathways such as AMPK. Exosomal miRNAs from melanoma cells exposed to IH differ in their cargo and effects on innate immune cells such as macrophages, and such differences are reflected by the melanoma cell type, with *STK11*-related pathways emerging as operational contributors to the altered melanoma phenotype induced by IH in vitro and by OSA in vivo.

## Figures and Tables

**Table 1:** List of KEGG pathways involved in 8172 unique gene target predictions found in 46 miRNAs in 1424 cells exposed to IH compared normoxia.

KEGG ID	KEGG Pathways	%	*p*-Value	Benjamini
sa05200	Pathways in cancer	2.598	2.92 × 10^−9^	2.598
hsa04810	Regulation of actin cytoskeleton	1.520	1.04 × 10^−8^	1.520
hsa05231	Choline metabolism in cancer	0.846	1.09 × 10^−8^	0.846
hsa04713	Ras signaling pathway	1.569	1.72 × 10^−7^	1.569
hsa04151	Circadian entrainment	0.772	2.75 × 10^−7^	0.772
hsa04152	PI3K-Akt signaling pathway	2.206	1.09 × 10^−6^	2.206
hsa04010	AMPK signaling pathway	0.907	5.42 × 10^−6^	0.907
hsa04070	Endocytosis	1.581	8.61 × 10^−6^	1.581
hsa04015	Phosphatidylinositol signaling system	0.748	9.53 × 10^−5^	0.748
hsa04010	MAPK signaling pathway	1.65	1.19 × 10^−5^	1.65
hsa04012	Rap1 signaling pathway	1.397	1.34 × 10^−5^	1.397
hsa04071	ErbB signaling pathway	0.674	1.50 × 10^−5^	0.674
hsa04120	Sphingolipid signaling pathway	0.870	1.92 × 10^−5^	0.870
hsa04921	Ubiquitin mediated proteolysis	0.968	2.20 × 10^−5^	0.968
hsa04919	Oxytocin signaling pathway	1.042	2.52 × 10^−5^	1.042
hsa04068	Thyroid hormone signaling pathway	0.833	3.04 × 10^−5^	0.833
hsa04150	FoxO signaling pathway	0.944	3.36 × 10^−5^	0.944
hsa04540	mTOR signaling pathway	0.478	5.00 × 10^−5^	0.478
hsa04024	Gap junction	0.662	5.93 × 10^−5^	0.662
hsa04261	cAMP signaling pathway	1.299	5.99 × 10^−5^	1.299
hsa05218	Adrenergic signaling	0.956	6.41 × 10^−5^	0.956
hsa05205	Melanoma	0.551	9.65 × 10^−5^	0.551
hsa04020	Proteoglycans in cancer	1.299	1.02 × 10^−5^	1.299
hsa04022	Calcium signaling pathway	1.176	1.28 × 10^−5^	1.176
hsa04666	cGMP-PKG signaling pathway	1.042	2.86 × 10^−5^	1.042
hsa04930	Fc gamma R-mediated phagocytosis	0.613	3.32 × 10^−5^	0.613
hsa03018	Type II diabetes mellitus	0.392	3.62 × 10^−5^	0.392
hsa04722	Prostate cancer	0.625	6.8 × 10^−5^	0.625
hsa04510	Neurotrophin signaling pathway	0.809	7.02 × 10^−4^	0.809
hsa05220	Focal adhesion	1.287	7.25 × 10^−4^	1.287
hsa05221	Chronic myeloid leukemia	0.527	8.68 × 10^−4^	0.527
hsa04520	Acute myeloid leukemia	0.429	1.13 × 10^−3^	0.429
hsa05212	Adherens junction	0.515	1.13 × 10^−3^	0.515
hsa04310	Pancreatic cancer	0.478	1.42 × 10^−3^	0.478
hsa00564	Wnt signaling pathway	0.895	1.49 × 10^−3^	0.895
hsa04931	Glycerophospholipid metabolism	0.650	1.75 × 10^−3^	0.650
hsa04931	Insulin resistance	0.723	1.74 × 10^−3^	0.723

**Table 2:** List of KEGG pathways involved in 2073 unique gene target predictions found in 8 miRNAs in 1675 cells exposed to IH compared normoxia.

KEGG ID	KEGG Pathways	%	*p*-Value	Benjamini
hsa05205	Proteoglycans in cancer	1.833	1.46 × 10^−4^	0.03
hsa05202	Transcriptional mis-regulation in cancer	1.586	2.37 × 10^−4^	0.03
hsa04024	cAMP signaling pathway	1.784	2.60 × 10^−4^	0.02
hsa04012	ErbB signaling pathway	0.991	4.87 × 10^−4^	0.03
hsa04010	MAPK signaling pathway	2.081	5.37 × 10^−4^	0.02
hsa04071	Sphingolipid signaling pathway	1.189	9.34 × 10^−4^	0.02

**Table 3:** Exosomes derived from melanoma 1424, and 1675 cells exposed to normoxia and hypoxia and applied into THP1 monocytes.

Cell Type	Genes	M1 Markers			Genes	M2 Markers		
		Normoxia	Hypoxia	*p*-Value		Normoxia	Hypoxia	*p*-Value
Exosomes from 1424 cells on monocytes	CCR7	1	0.90 ± 0.06	0.541	CD206	1	0.83 ± 0.05	0.542
	CXCL10	1	1.28 ± 0.014	0.023	CD163	1	1.17 ± 0.016	0.421
	IL6	1	1.21 ± 0.013	0.014	IL10	1	1.11 ± 0.011	0.621
**Cell Type**	**Genes**	**M1 Markers**			**Genes**	**M2 Markers**		
		**Normoxia**	**Hypoxia**	***p*-Value**		**Normoxia**	**Hypoxia**	***p*-Value**
Exosomes from 1675 cells on monocytes	CCR7	1	1.75 ± 0.013	0.012	CD206	1	1.21 ± 0.014	0.023
	CXCL10	1	1.52 ± 0.014	0.024	CD163	1	1.86 ± 0.016	0.045
	IL6	1	1.77 ± 0.015	0.004	IL10	1	1.44 ± 0.014	0.041

**Table 4:** Exosomes derived from melanoma 1424, and 1675 cells exposed to normoxia and intermittent hypoxia and applied into THP1 macrophages.

Cell Type	Genes	M1 Markers			Genes	M2 Markers		
		Normoxia	Hypoxia	*p*-Value		Normoxia	Hypoxia	*p*-Value
Exosomes from 1424 cells on macrophages	CCR7	1	1.11 ± 0.012	0.121	CD206	1	1.02 ± 0.01	0.091
	CXCL10	1	1.53 ± 0.016	0.001	CD163	1	0.98 ± 0.07	0.131
	IL6	1	0.93 ± 0.06	0.071	IL10	1	0.91 ± 0.08	0.082
**Cell Type**	**Genes**	**M1 Markers**			**Genes**	**M2 Markers**		
		**Normoxia**	**Hypoxia**	***p*-Value**		**RA**	**IH**	***p*-Value**
Exosomes from 1675 cells on macrophages	CCR7	1	1.26 ± 0.016	0.042	CD206	1	1.01 ± 0.00	0.122
	CXCL10	1	1.35± 0.014	0.022	CD163	1	1.06 ± 0.00	0.092
	IL6	1	1.12 ± 0.011	0.061	IL10	1	1.00 ± 0.00	0.211

## Data Availability

The data presented in this study are available on request from the corresponding author. The data are not publicly available due to privacy and other bioethical considerations.

## Supplementary Materials

The following are available online at https://www.mdpi.com/article/10.3390/cancers13194781/s1, Figure S1: Characterization and quantification of plasma derived exosomes from OSA-V1 and OSA-V2 subjects, Figure S2: Confocal microscope images illustrating exosomes derived from OSA up taken by naïve human melanoma cells, Figure S3: THP-1 cells displayed altered expression of CD11b when monocytes were differentiated into macrophages by PMA as described in the Materials and Methods section, Figure S4: Western blot figure, Table S1A: List of most significant gene ontology related genes including cellular component involved in 8172 unique gene target predictions found in 46 miRNAs in 1424 cells exposed to IH compared to normoxia, Table S1B: List of most significant gene ontology including biological process involved in 8172 unique gene target predictions found in 46 miRNAs in 1424 cells exposed to IH compared to normoxia, Table S1C: List of most significant gene ontology including molecular function involved in 8172 unique target gene predictions found in 46 miRNAs in 1424 cells exposed to IH compared to normoxia., Table S2A: List of most significant gene ontology including cellular component involved in 2073 unique gene target predictions found in 8 miRNAs in 1675 cells exposed to IH compared normoxia, Table S2B: List of most significant gene ontology including biological process involved in 2073 unique gene target predictions found in 8 miRNAs in 1675 cells exposed to IH compared normoxia.
