# Supplementary material for: Heterogeneity of Melanoma Cell Responses to Sleep Apnea-Derived Plasma Exosomes and to Intermittent Hypoxia
Source: Cancers (Basel). 2021 Sep 24;13(19):4781. doi: 10.3390/cancers13194781 (PMC8508428; doi:10.3390/cancers13194781)
Supplement: Supplementary file 1 [file cancers-13-04781-s001.zip › cancers-1385487-supplementary.pdf]

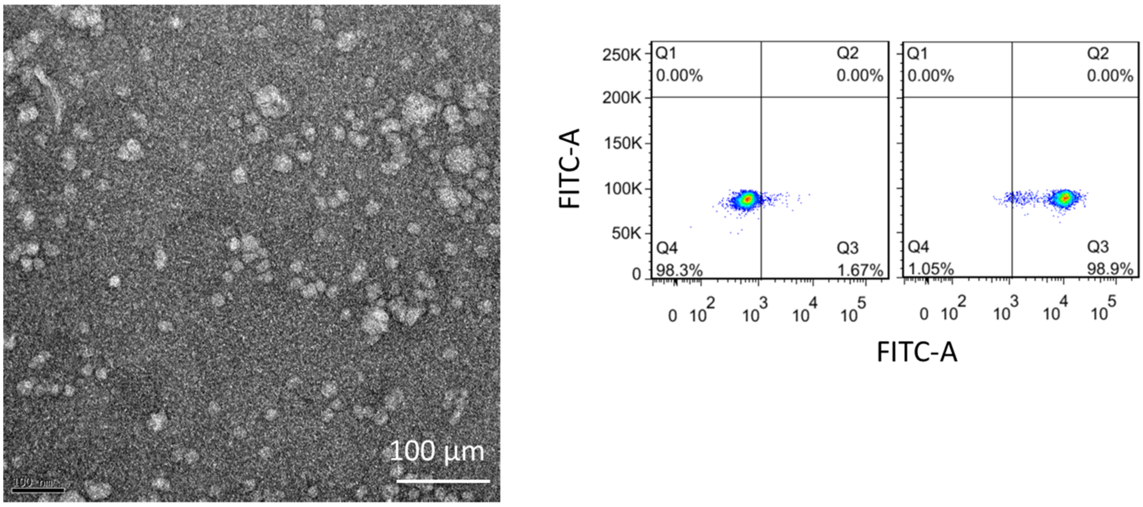

(**A**) (**B**)

Figure S1. Characterization and quantification of plasma derived exosomes from OSA-V1 and OSA-V2 subjects. (**A**) Representative electron microscope image for isolated exosomes, scale bar 100 nm. (**B**) Flow cytometry analyses for exosome surface markers including tetraspanin (CD63) marker in the presence or absence of exosomes were analyzed using a FACS Calibur instrument. Bead flow separation data for exosomes were analyzed using the various capture antibodies followed by Exo-FITC staining, and independent experiments were repeated 4 times. The FITC flow cytometric intensities were then plotted versus the number of exosome particles input into the flow reaction. The data are graphed showing forward scatter versus FITC intensity (n=6).

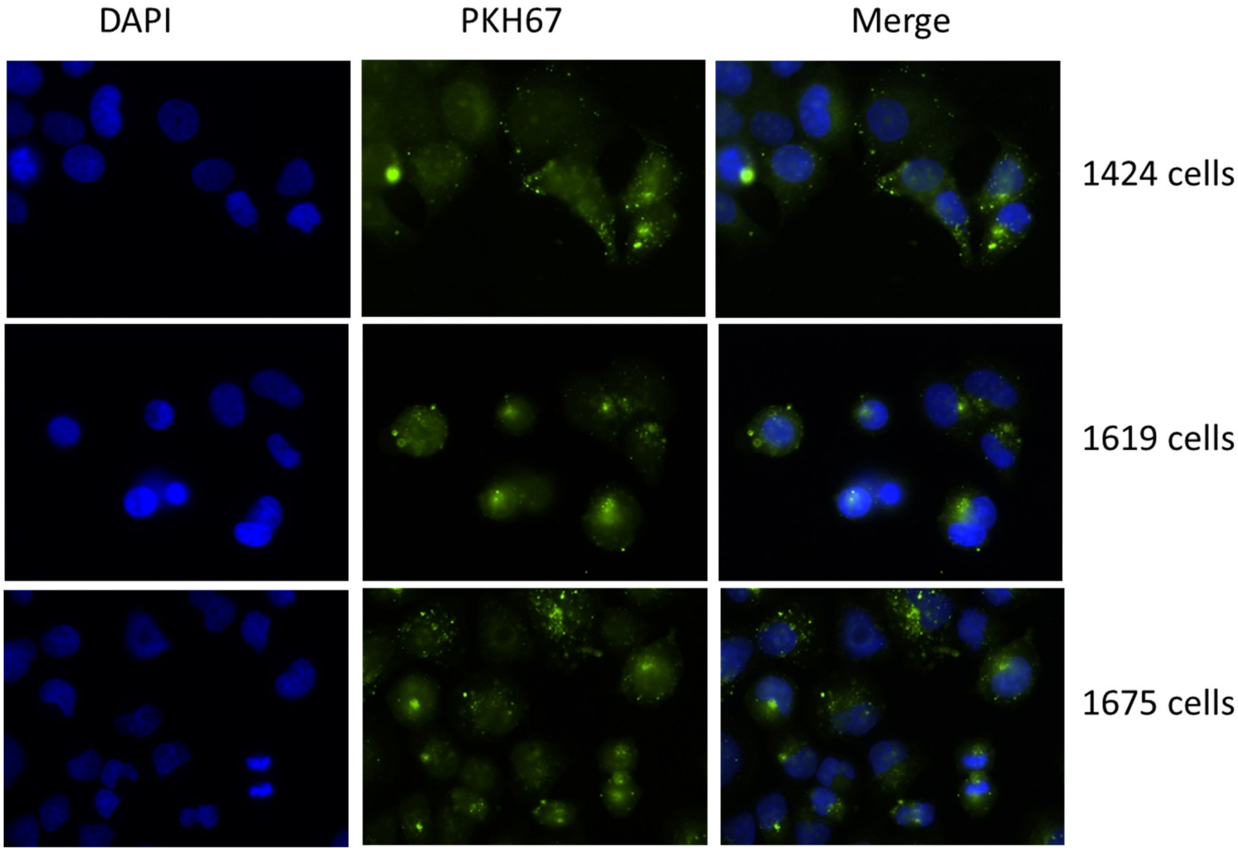

Figure S2: Confocal microscope images illustrating exosomes derived from OSA up taken by naïve human melanoma cells. Exosomes from plasma of OSA-V1 and OSA-V2 were labeled with a PKH67-Green Fluorescent Cell Linker Kit. Human melanoma cells were grown on coverslips for 24 h, and the PKH67-labeled exosomes from OSA-V1 and OSA-V2 were added to the melanoma cells at 37°C for 24 h. Cells were washed and stained with nuclei (blue) stained with DAPI. As controls, no exosomes were used but PKH26 was added to the cell medium. Exosome uptake was measured using confocal laser scanning microscopy (LSP5) at 490 nm excitation and 502 nm emission (n = 6; scale bar: 10 µm).

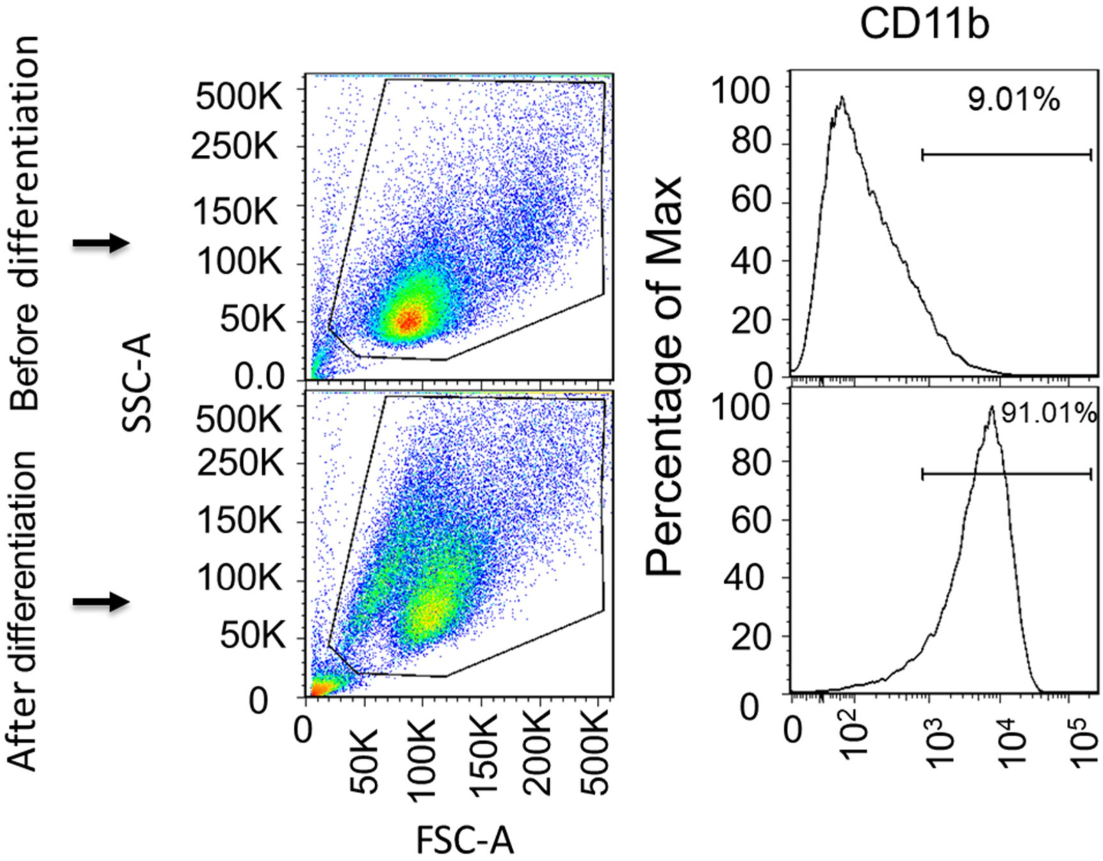

Figure S3. THP-1 cells displayed altered expression of CD11b when monocytes were differentiated into macrophages by PMA as described in the Materials and Methods section. Cells were stained with CD11b antibody and THP-1 cells were quantified by flow cytometry. A representative histogram of three independent experiments is displayed.

Table S1. A: List of most significant gene ontology including cellular component involved in 8172 unique target predications genes found in 46 miRNAs in 1424 cells exposed to IH compared to normoxia.

Cellular Component ID	Term	%	*p* Value	Benjamini
GO:0005654	nucleoplasm	17.63	1.81E-10	2.20E-07
GO:0044428	nuclear part	22.95	5.06E-10	3.07E-07
GO:0097060	synaptic membrane	2.06	2.07E-09	8.40E-07
GO:0031981	nuclear lumen	20.74	3.65E-09	1.11E-06
GO:0005634	nucleus	39.13	1.47E-08	1.51E-06
GO:0000139	Golgi membrane	4.53	4.25E-08	8.60E-06
GO:0098590	plasma membrane region	5.77	1.84E-06	2.79E-04
GO:0005794	Golgi apparatus	8.82	2.35E-06	3.17E-04
GO:0043229	intracellular organelle	64.98	3.56E-06	2.17E-04
GO:0045211	postsynaptic membrane	1.51	1.06E-05	0.001166535
GO:0005737	cytoplasm	58.05	1.77E-05	0.000116653
GO:0044431	Golgi apparatus part	5.53	2.18E-05	0.001894282
GO:0043231	intracellular membrane-bounded organelle	59.35	3.13E-05	0.000216653
GO:0098797	plasma membrane protein complex	3.33	3.53E-05	0.002680256

GO:0036464	cytoplasmic ribonucleoprotein granule	1.08	4.64E-05	0.002812351
GO:0030672	synaptic vesicle membrane	0.53	6.54E-05	0.003774321
GO:0099501	exocytic vesicle membrane	0.53	6.54E-05	0.003774321
GO:0070382	exocytic vesicle	1.03	8.92E-05	0.004913701
GO:0035770	ribonucleoprotein granule	1.13	1.28E-04	0.006722612
GO:0034702	ion channel complex	1.89	1.68E-04	0.008458595
GO:0016604	nuclear body	2.30	2.32E-04	0.011215156
GO:0015630	microtubule cytoskeleton	6.47	2.71E-04	0.012583619
GO:0033267	axon part	1.47	2.99E-04	0.01334978
GO:0000118	histone deacetylase complex	0.51	3.05E-04	0.013170595
GO:0044853	plasma membrane raft	0.64	3.18E-04	0.013257284
GO:0070603	SWI/SNF superfamily-type complex	0.59	4.21E-04	0.016922863
GO:0044451	nucleoplasm part	4.45	4.43E-04	0.017211109
GO:0098802	plasma membrane receptor complex	1.20	5.17E-04	0.019454072
GO:0090545	CHD-type complex	0.20	5.29E-04	0.019276794
GO:0016581	NuRD complex	0.20	5.29E-04	0.019276794
GO:0090568	nuclear transcriptional repressor complex	0.23	5.97E-04	0.021101325
GO:1902495	transmembrane transporter complex	2.06	9.12E-04	0.031163261
GO:0005802	trans-Golgi network	1.34	9.26E-04	0.030774949
GO:0009898	cytoplasmic side of plasma membrane	1.10	9.63E-04	0.031157822
GO:0055037	recycling endosome	0.97	0.001083	0.03406217
GO:0008287	protein serine/threonine phosphatase complex	0.40	0.00126	0.038506442
GO:0070013	intracellular organelle lumen	23.79	0.00127	0.037506442
GO:0030658	transport vesicle membrane	1.08	0.001282	0.03729073
GO:0000932	cytoplasmic mRNA processing body	0.59	0.001444	0.040948571
GO:0030133	transport vesicle	2.17	0.001475	0.040845451
GO:0005829	cytosol	18.79	0.00179	0.048273906
GO:0048471	perinuclear region of cytoplasm	3.81	0.001894	0.049892377

Table S1. B: List of most significant gene ontology including biological process involved in 8172 unique target predications genes found in 46 miRNAs in 1424 cells exposed to IH compared to normoxia.

Biological Process ID	Biological Process Term	%	*p* Value	Benjamin
GO:0007399	nervous system development	13.66421569	2.39E-23	1.80E-19
GO:0036211	protein modification process	22.59803922	3.50E-22	1.31E-18
GO:0006464	cellular protein modification process	22.59803922	3.50E-22	1.31E-18
GO:0048468	cell development	12.35294118	1.79E-20	4.48E-17
GO:2000112	regulation of cellular macromolecule biosynthetic process	23.21078431	1.82E-18	3.43E-15
GO:0030182	neuron differentiation	8.014705882	3.54E-18	5.32E-15
GO:0051252	regulation of RNA metabolic process	21.91176471	1.29E-17	1.21E-14
GO:0022008	neurogenesis	9.215686275	1.46E-16	9.26E-14
GO:0006355	regulation of transcription, DNA-templated	20.89460784	3.28E-15	2.50E-12
GO:0007417	central nervous system development	6.004901961	5.30E-15	3.64E-12
GO:0010604	positive regulation of macromolecule metabolic process	16.83823529	9.34E-15	5.84E-12
GO:0045935	positive regulation of compound metabolic process	10.26960784	9.99E-15	5.77E-12
GO:2001141	regulation of RNA biosynthetic process	21.06617647	1.02E-14	5.48E-12
GO:0045893	positive regulation of transcription, DNA-templated	8.517156863	1.91E-14	9.56E-12
GO:0048666	neuron development	6.323529412	2.30E-14	1.08E-11
GO:0051254	positive regulation of RNA metabolic process	8.87254902	3.39E-14	1.50E-11
GO:0035556	intracellular signal transduction	15.71078431	3.53E-14	1.47E-11
GO:0010557	positive regulation of macromolecule biosynthetic process	9.87745098	5.46E-14	2.16E-11
GO:0006351	transcription, DNA-templated	20.73529412	1.84E-13	6.90E-11
GO:0009887	organ morphogenesis	6.421568627	2.28E-13	8.14E-11
GO:1902680	positive regulation of RNA biosynthetic process	8.541666667	4.11E-13	1.40E-10
GO:0016569	covalent chromatin modification	3.823529412	5.44E-13	1.78E-10
GO:0010628	positive regulation of gene expression	10.31862745	8.24E-13	2.58E-10
GO:0000902	cell morphogenesis	7.781862745	1.34E-12	4.04E-10
GO:0031175	neuron projection development	5.367647059	1.56E-12	4.52E-10
GO:0031328	positive regulation of cellular biosynthetic process	10.49019608	2.19E-12	6.10E-10
GO:0007420	brain development	4.583333333	2.53E-12	6.77E-10

GO:0048513	animal organ development	18.06372549	3.67E-12	9.50E-10
GO:0032990	cell part morphogenesis	5.306372549	1.90E-11	4.75E-09
GO:0048858	cell projection morphogenesis	5.183823529	2.56E-11	6.21E-09
GO:0032774	RNA biosynthetic process	22.08333333	3.60E-11	8.44E-09
GO:0050804	modulation of synaptic transmission	2.095588235	6.79E-11	1.54E-08
GO:0090304	nucleic acid metabolic process	28.62745098	6.79E-11	1.56E-08
GO:0007167	enzyme linked receptor protein signaling	6.053921569	6.92E-11	1.49E-08
GO:0009966	regulation of signal transduction	15.77205882	9.31E-11	1.94E-08
GO:0048812	neuron projection morphogenesis	3.676470588	9.62E-11	1.95E-08
GO:0034645	cellular macromolecule biosynthetic process	27.83088235	1.22E-10	1.98E-08
GO:0000904	cell morphogenesis involved in differentiation	4.87745098	2.12E-10	4.09E-08
GO:2000113	negative regulation of cellular macromolecule	8.088235294	2.55E-10	4.79E-08
GO:0051960	regulation of nervous system development	4.963235294	2.68E-10	4.90E-08
GO:0050767	regulation of neurogenesis	4.460784314	2.92E-10	5.22E-08
GO:0072358	cardiovascular system development	5.943627451	5.59E-10	9.77E-08
GO:0072359	circulatory system development	5.943627451	5.59E-10	9.77E-08
GO:0016358	dendrite development	1.495098039	5.92E-10	1.01E-07
GO:0010558	negative regulation of macromolecule biosynthetic process	8.553921569	1.22E-09	2.04E-07
GO:0034654	nucleobase-containing compound biosynthetic process	24.27696078	1.23E-09	2.05E-07
GO:0045664	regulation of neuron differentiation	3.713235294	1.58E-09	2.53E-07
GO:0031327	negative regulation of cellular biosynthetic process	8.884803922	1.62E-09	2.53E-07

Table S1. C: List of most significant gene ontology including molecular function involved in 8172 unique target predications genes found in 46 miRNAs in 1424 cells exposed to IH compared to normoxia.

Molecular Function ID	Molecular Function Term	%	*p* Value	Benjamini
GO:0000975	regulatory region DNA binding	5.613	1.44E-12	1.55E-09
GO:0003690	double-stranded DNA binding	5.306	2.31E-12	1.24E-09
GO:0032555	purine ribonucleotide binding	11.275	3.06E-11	8.24E-09
GO:0004672	protein kinase activity	4.350	3.86E-11	8.31E-09
GO:0017076	purine nucleotide binding	11.324	5.67E-11	1.02E-08
GO:0032550	purine ribonucleoside binding	11.066	5.70E-11	8.76E-09
GO:0032553	ribonucleotide binding	11.324	8.76E-11	1.18E-08
GO:0032559	adenyl ribonucleotide binding	9.265	2.22E-10	2.66E-08
GO:0030554	adenyl nucleotide binding	9.314	3.08E-10	3.32E-08
GO:0005524	ATP binding	9.044	4.17E-10	4.08E-08
GO:0043565	sequence-specific DNA binding	6.703	1.15E-09	1.03E-07
GO:0061630	ubiquitin protein ligase activity	1.422	2.77E-06	2.29E-04
GO:0031267	small GTPase binding	1.973	3.66E-06	2.81E-04
GO:0031625	ubiquitin protein ligase binding	1.949	3.81E-06	2.73E-04
GO:0019901	protein kinase binding	3.309	1.94E-05	0.001302
GO:0035004	phosphatidylinositol 3-kinase activity	0.576	4.75E-05	0.003004
GO:0052813	phosphatidylinositol bisphosphate kinase activity	0.527	1.78E-04	0.010576
GO:0000287	magnesium ion binding	1.360	3.20E-04	0.017966
GO:0008017	microtubule binding	1.446	3.42E-04	0.018243
GO:0046914	transition metal ion binding	8.051	3.57E-04	0.018131
GO:0008066	glutamate receptor activity	0.257	7.06E-04	0.033938
GO:0052742	phosphatidylinositol kinase activity	0.417	8.33E-04	0.03822
	(1)			

Table S2. A: List of most significant gene ontology including cellular component involved in 2073 unique target predications genes found in 8 miRNAs in 1675 cells exposed to IH compared normoxia.

Cellular Components ID	Term	%	*p* Value	Benjamini
GO:0014069	GO:0014069~postsynaptic density	2.1308	2.19E-06	0.0017163
GO:0099572	GO:0099572~postsynaptic specialization	2.1308	2.19E-06	0.0017163
GO:0005634	GO:0005634~nucleus	42.1705	7.69E-06	0.0030104
GO:0044309	GO:0044309~neuron spine	1.4371	1.25E-05	0.0032585
GO:0043197	GO:0043197~dendritic spine	1.3875	2.52E-05	0.0049209
GO:0097060	GO:0097060~synaptic membrane	2.6759	2.55E-05	0.0039916

GO:0030424	GO:0030424~axon	3.5679	8.65E-05	0.0112413
GO:0030425	GO:0030425~dendrite	3.8652	1.13E-04	0.012566

Table S2. B: List of most significant gene ontology including biological process involved in 2073 unique target predications genes found in 8 miRNAs in 1675 cells exposed to IH compared normoxia.

Biological Process ID+B2:F21	Biological Process Term	%	*p* Value	Bonferroni
GO:0010468	regulation of gene expression	29.386	1.40E-15	7.04E-12
GO:0051252	regulation of RNA metabolic process	25.818	1.70E-14	8.29E-11
GO:0006355	regulation of transcription, DNA-templated	24.381	1.85E-12	9.02E-09
GO:0006351	transcription, DNA-templated	24.232	6.59E-12	3.22E-08
GO:2000112	regulation of cellular macromolecule biosynthetic process	26.412	6.62E-12	3.23E-08
GO:2001141	regulation of RNA biosynthetic process	24.430	7.90E-12	3.86E-08
GO:0010556	regulation of macromolecule biosynthetic process	26.957	1.36E-11	6.62E-08
GO:0032774	RNA biosynthetic process	25.768	2.32E-11	1.13E-07
GO:0007399	nervous system development	15.709	4.03E-11	1.97E-07
GO:0016070	RNA metabolic process	29.286	1.65E-09	8.07E-06
GO:0036211	protein modification process	24.678	2.07E-09	1.01E-05
GO:0006464	cellular protein modification process	24.678	2.07E-09	1.01E-05
GO:0034654	nucleobase-containing compound biosynthetic process	27.552	4.24E-09	2.07E-05
GO:0034645	cellular macromolecule biosynthetic process	31.120	4.24E-09	2.07E-05
GO:0090304	nucleic acid metabolic process	31.467	6.53E-08	3.19E-04
GO:0043549	regulation of kinase activity	6.095	3.94E-06	0.0190412
GO:0022008	neurogenesis	10.059	5.10E-06	0.0246064
GO:0048468	cell development	13.033	7.29E-06	0.034936
GO:0010604	positive regulation of macromolecule metabolic process	18.038	7.76E-06	0.0371451

Table S2. C: List of most of significant gene ontology including molecular function involved in 2073 unique target predications genes found in 8 miRNAs in 1675 cells exposed to IH compared normoxia.

Molecular Function ID	Molecular Function Term	%	*p* Value	Benjamini
GO:0000975	regulatory region DNA binding	7.185332	7.55E-09	4.18E-06
GO:0003690	double-stranded DNA binding	6.838454	8.49E-09	2.35E-06
GO:0043565	sequence-specific DNA binding	8.473736	4.15E-08	7.67E-06
GO:0004672	protein kinase activity	5.450942	7.98E-07	1.11E-04
GO:0001077	transcriptional activator activity	2.477701	3.05E-06	3.38E-04
