# Supplementary figures and images for: Heterogeneity of Melanoma Cell Responses to Sleep Apnea-Derived Plasma Exosomes and to Intermittent Hypoxia
Source: Cancers (Basel). 2021 Sep 24;13(19):4781. doi: 10.3390/cancers13194781 (PMC8508428; doi:10.3390/cancers13194781)

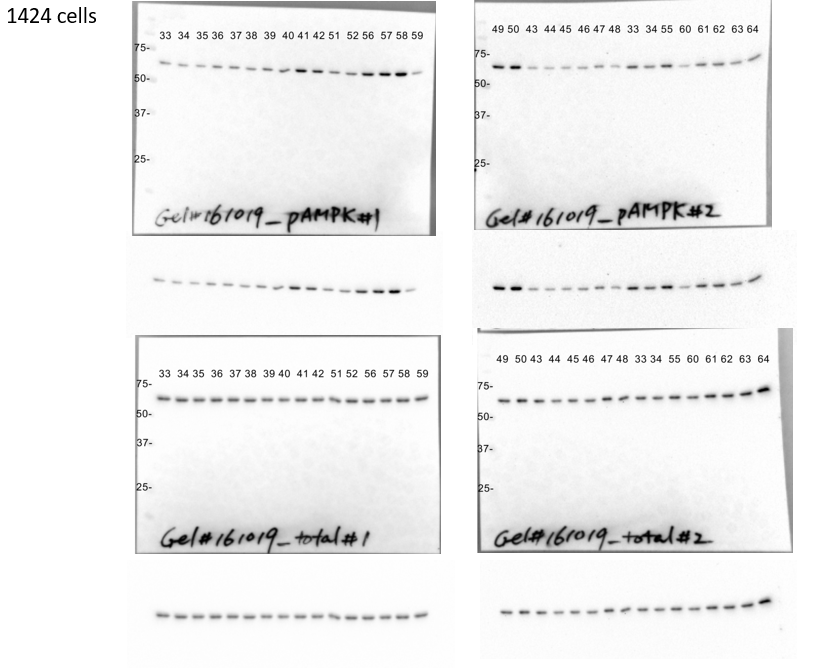

Supplement: Supplementary file 1 [file cancers-13-04781-s001.zip › Western blot figure 1.PNG]

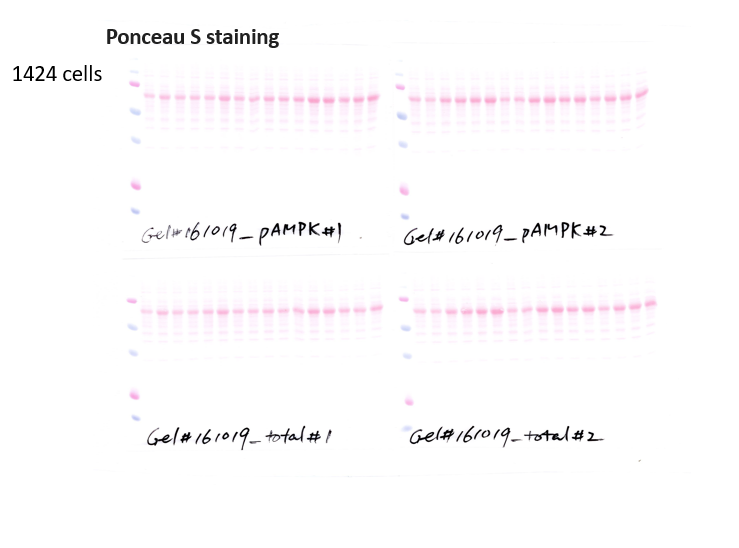

Supplement: Supplementary file 1 [file cancers-13-04781-s001.zip › Western blot figure 2.PNG]

## Slide 1
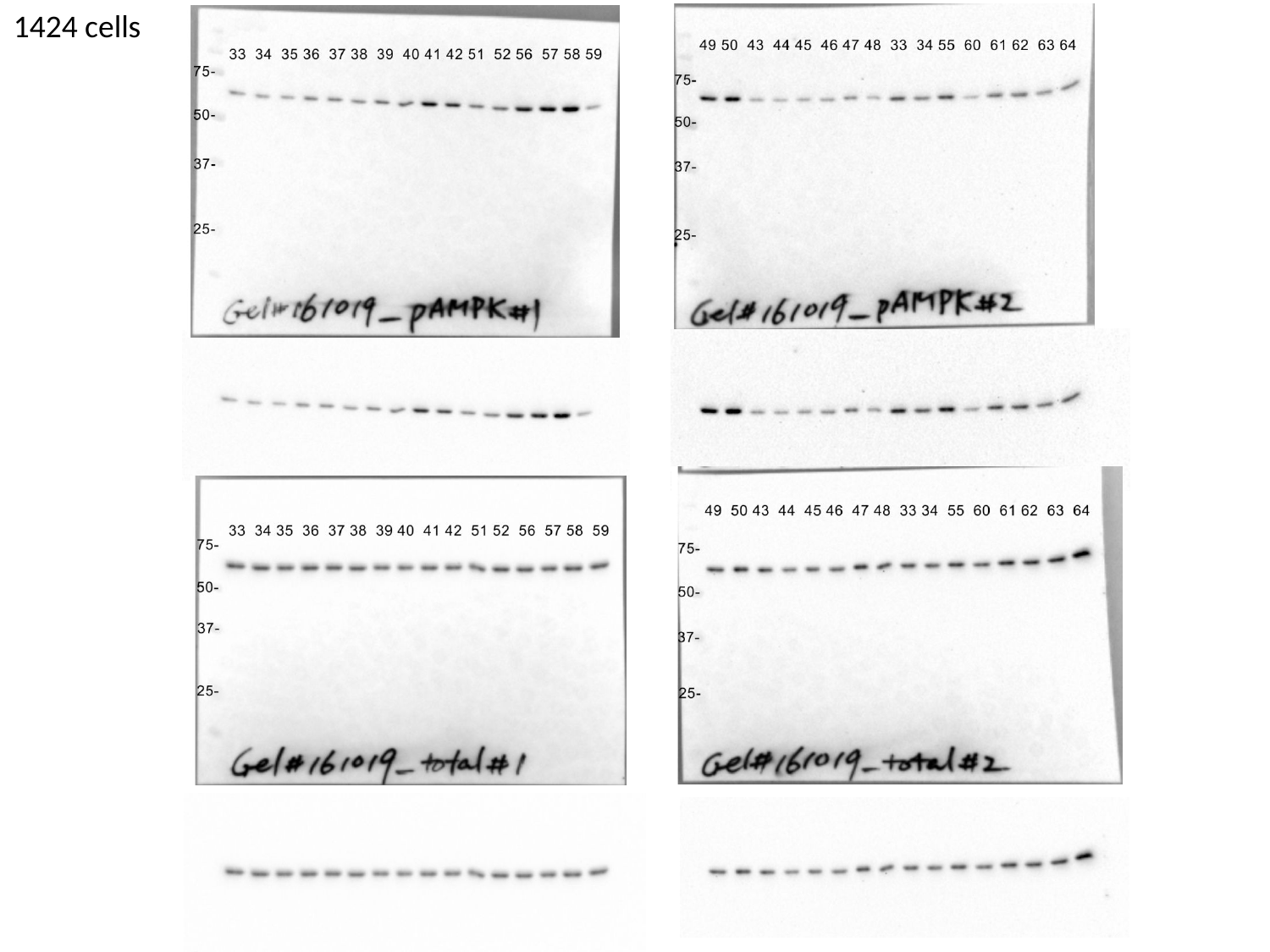

1424 cells

## Slide 2
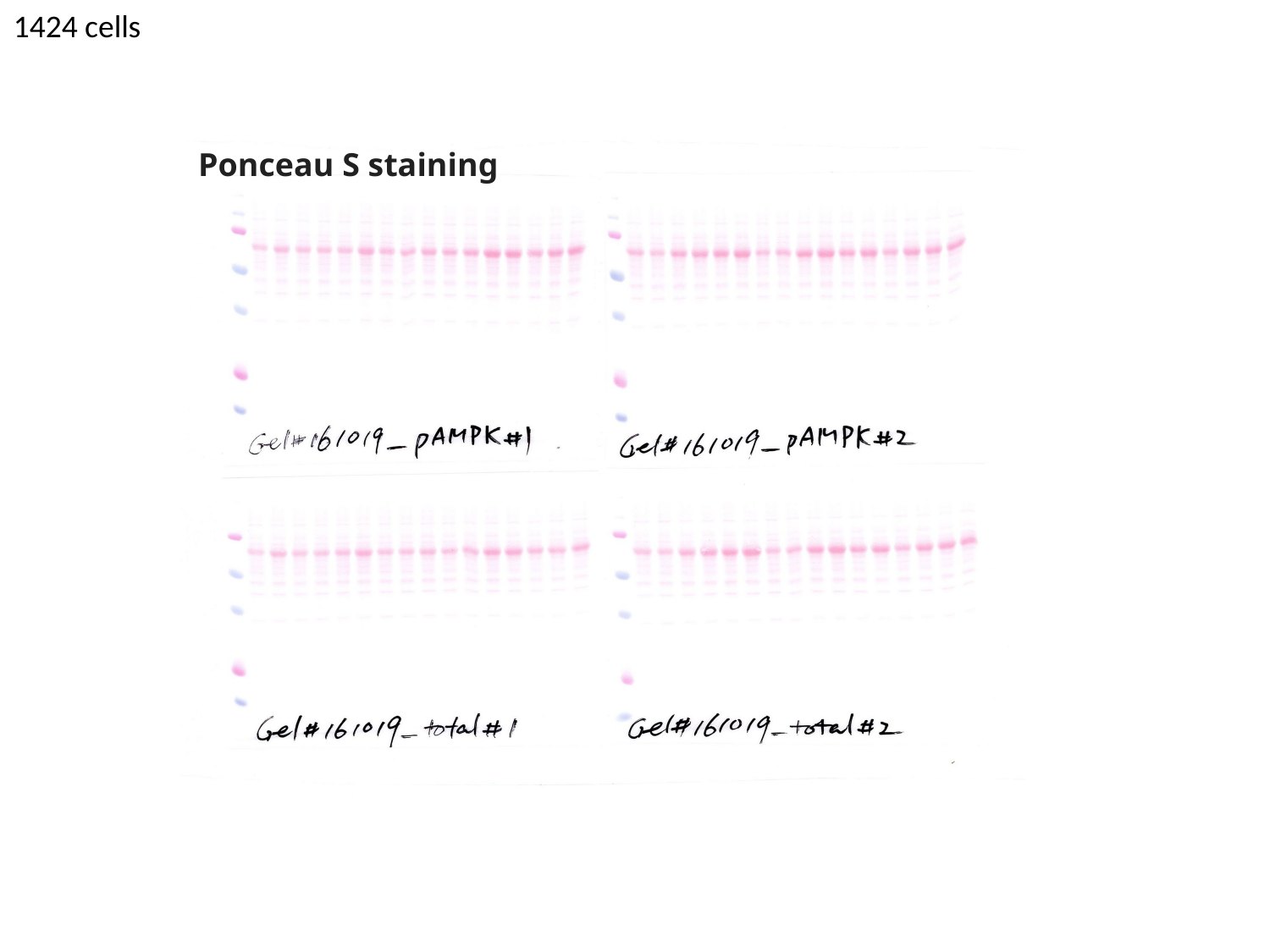

1424 cells
Ponceau S staining

Supplement: Supplementary file 1 [file cancers-13-04781-s001.zip › Western blot figures.pptx]

## Slide 1
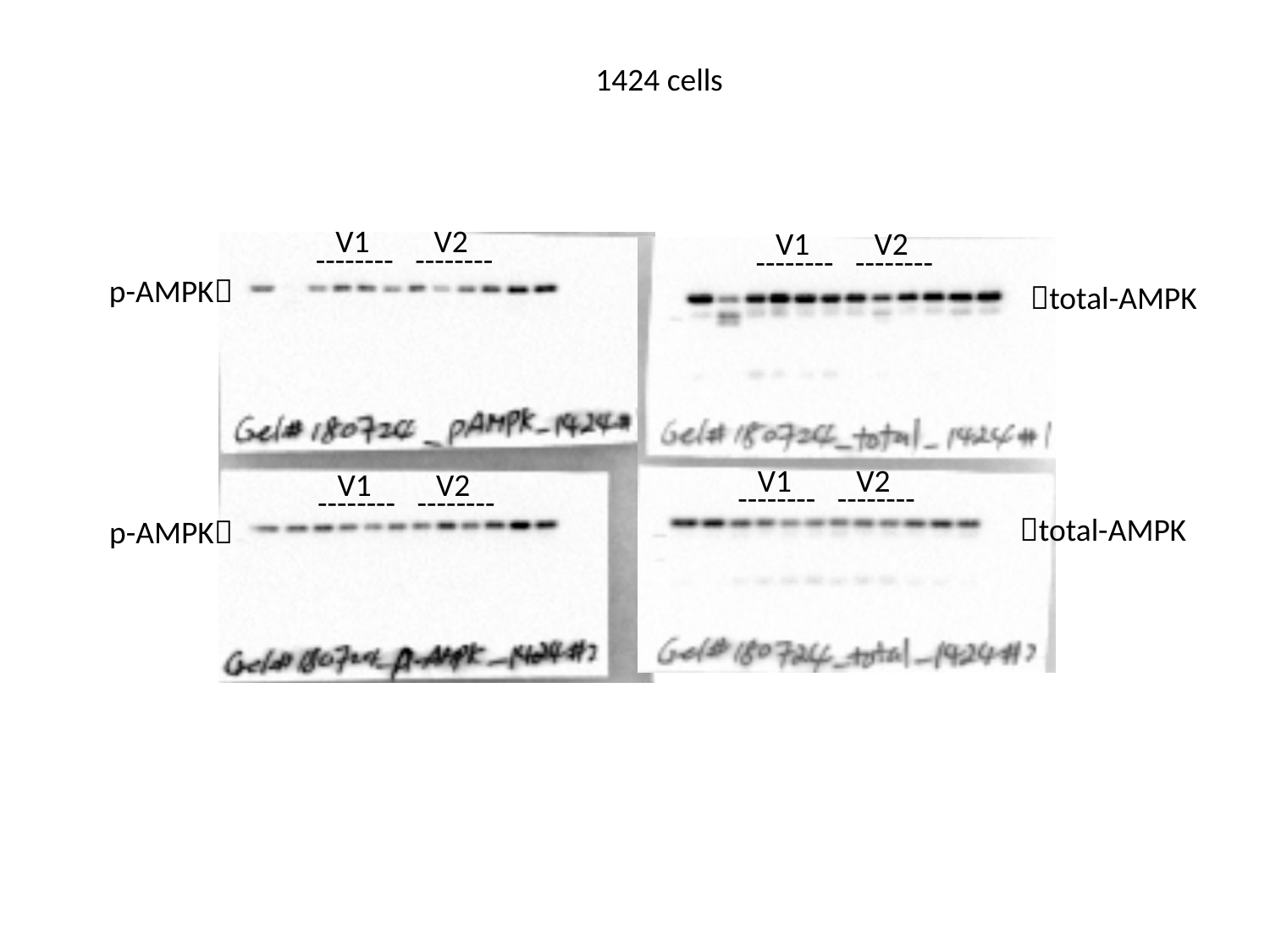

1424 cells
V1 V2
V1 V2
-------- --------
-------- --------
p-AMPK
total-AMPK
V1 V2
V1 V2
-------- --------
-------- --------
total-AMPK
p-AMPK

## Slide 2
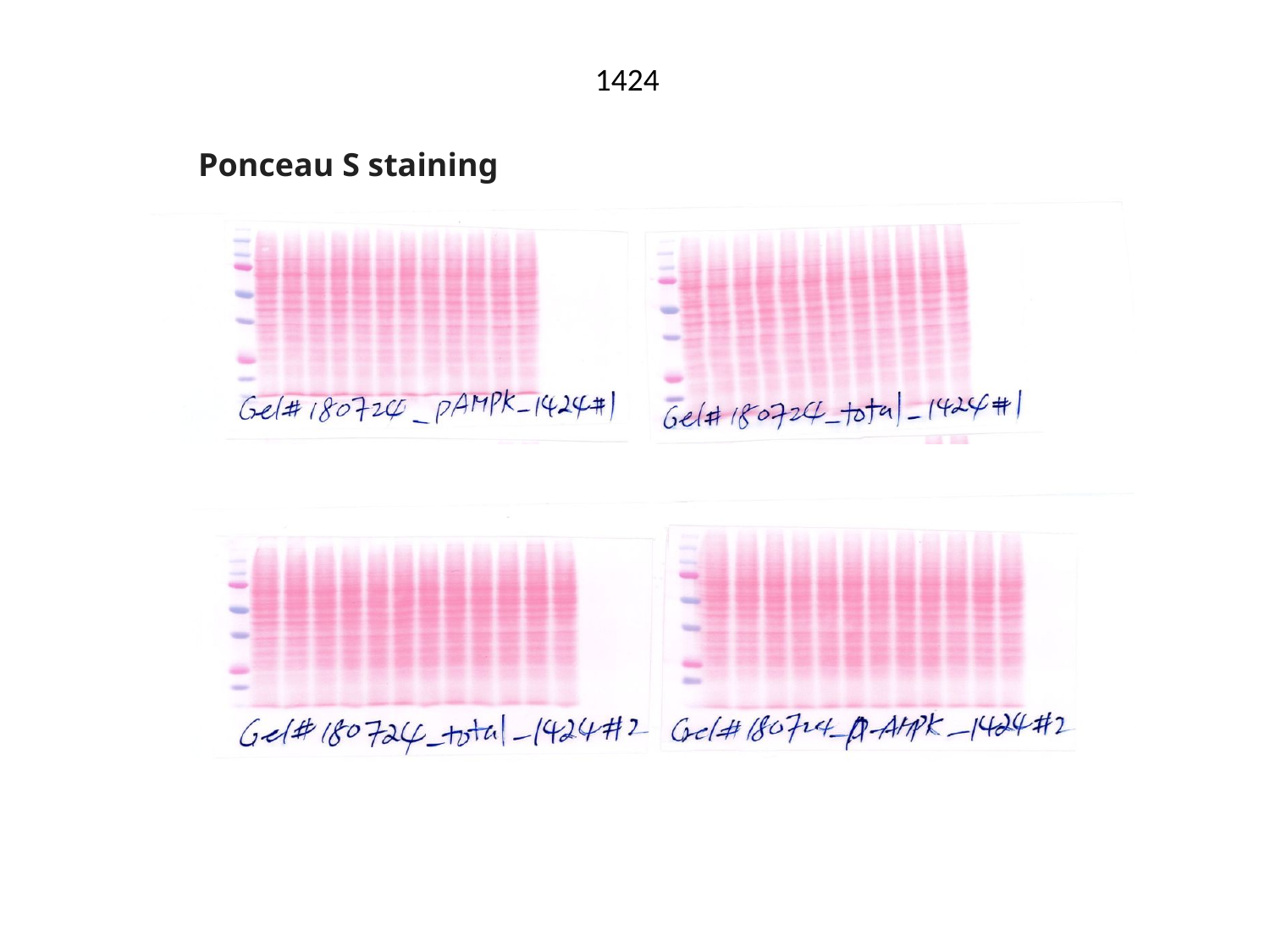

1424
Ponceau S staining

Supplement: Supplementary file 1 [file cancers-13-04781-s001.zip › Western blot figures2.pptx]
